# Involvement of inflammasomes in tumor microenvironment and tumor therapies

**DOI:** 10.1186/s13045-023-01407-7

**Published:** 2023-03-17

**Authors:** Ziqi Zhang, Xue Li, Yang Wang, Yuquan Wei, Xiawei Wei

**Affiliations:** Laboratory of Aging Research and Cancer Drug Target, State Key Laboratory of Biotherapy, National Clinical Research Center for Geriatrics, West China Hospital, Sichuan University, No. 17, Block 3, Southern Renmin Road, Chengdu, 610041 Sichuan People’s Republic of China

**Keywords:** Inflammasome, NLRP3, Pyroptosis, Tumor microenvironment, Immunity

## Abstract

Inflammasomes are macromolecular platforms formed in response to damage-associated molecular patterns (DAMPs) and pathogen-associated molecular patterns, whose formation would cause maturation of interleukin-1 (IL-1) family members and gasdermin D (GSDMD), leading to IL-1 secretion and pyroptosis respectively. Several kinds of inflammasomes detecting different types of dangers have been found. The activation of inflammasomes is regulated at both transcription and posttranscription levels, which is crucial in protecting the host from infections and sterile insults. Present findings have illustrated that inflammasomes are involved in not only infection but also the pathology of tumors implying an important link between inflammation and tumor development. Generally, inflammasomes participate in tumorigenesis, cell death, metastasis, immune evasion, chemotherapy, target therapy, and radiotherapy. Inflammasome components are upregulated in some tumors, and inflammasomes can be activated in cancer cells and other stromal cells by DAMPs, chemotherapy agents, and radiation. In some cases, inflammasomes inhibit tumor progression by initiating GSDMD-mediated pyroptosis in cancer cells and stimulating IL-1 signal-mediated anti-tumor immunity. However, IL-1 signal recruits immunosuppressive cell subsets in other cases. We discuss the conflicting results and propose some possible explanations. Additionally, we also summarize interventions targeting inflammasome pathways in both preclinical and clinical stages. Interventions targeting inflammasomes are promising for immunotherapy and combination therapy.

## Background

**Fig. 1:**
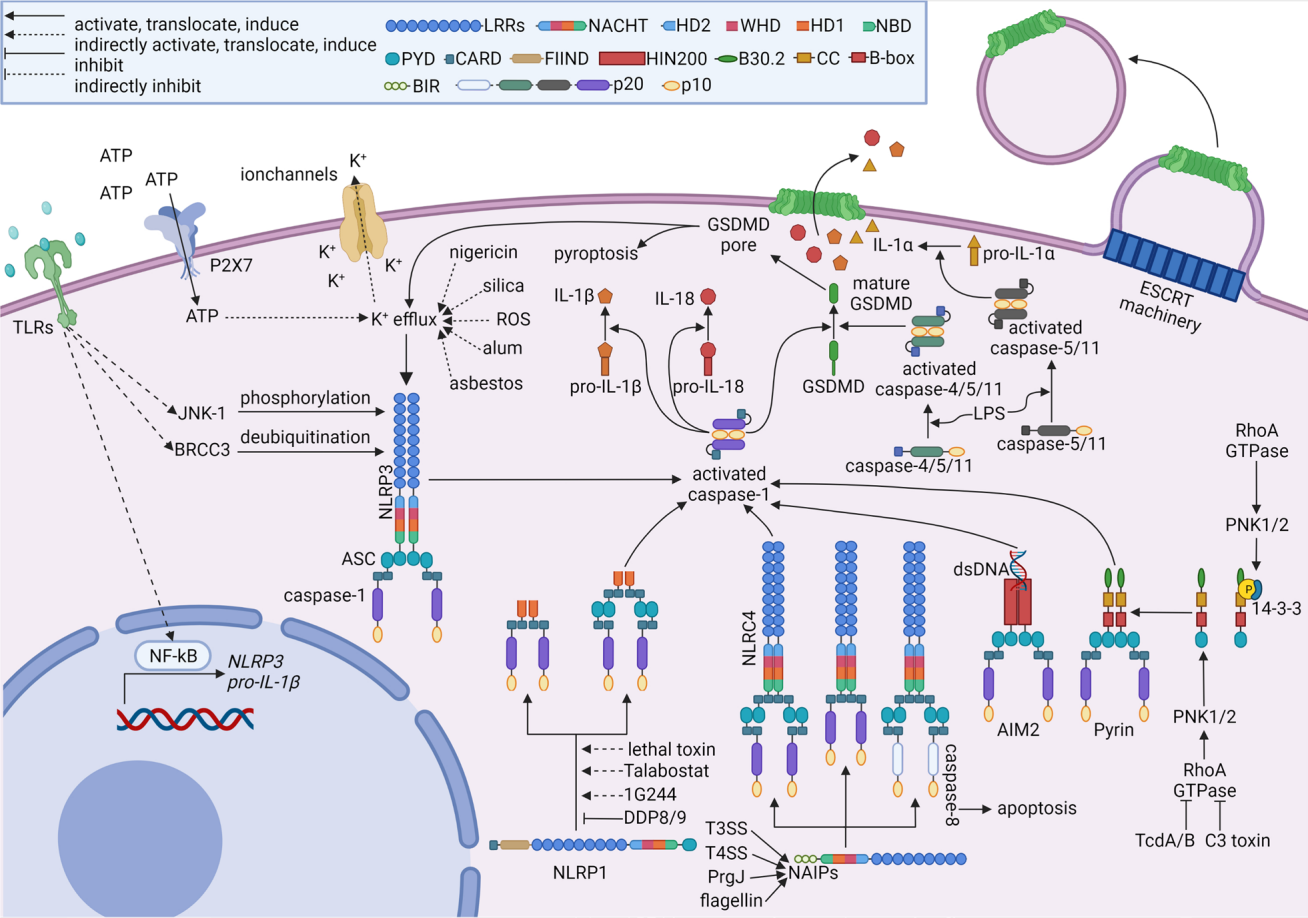
Overview of inflammasome activation

One of the crucial functions of the innate immune system is to recognize DAMPs and PAMPs by pattern recognition receptors (PRRs) during microbial infection and sterile damage [1]. Some PRRs, such as Toll-like receptors (TLRs), are located in the cytoplasm membrane and endosome membrane to supervise extracellular and endosomal dangers [2]. In the cytosol, nucleotide-binding leucine-rich repeat receptors (NLRs), absent in melanoma 2 (AIM2), and pyrin are able to recognize cytosolic DAMPs and PAMPs [3]. Distinct from TLRs that eventually elevate pro-inflammatory cytokines, type I interferons, and chemokines at the transcription level, NLRs (NACHT, leucine-rich repeat and pyrin domain-containing 1 (NLRP1), NOD-, LRR- and pyrin domain-containing 3 (NLRP3), and NLR family apoptosis inhibitory protein (NAIP)/NLR family CARD domain-containing 4 (NLRC4)), AIM2, and pyrin initiate posttranslational mechanisms by assembling inflammasomes, a group of multicomponent complexes [4, 5]. Briefly, inflammasome sensors recruit caspase-1 family members with or without the assistance of apoptosis-associated speck-like protein-containing CARD (ASC) to initiate auto-cleavage of caspase-1. The activated caspase-1 cleaves precursors of GSDMD and IL-1 family members to release these cytokines and induce pyroptosis. The canonical and non-canonical inflammasome pathways are summarized in Fig. 1.

Canonical inflammasomes are composed of sensors, ASC, and caspase-1 [5]. Once activated, these inflammasome sensors oligomerize and recruit ASC to form an “ASC speck” through pyrin–pyrin (PYD–PYD) interaction [6, 7]. Then caspase-1 is recruited to ASC through CARD–CARD interaction [6, 7]. However, exceptions have been reported in the activation of NLRP1 and NLRC4. CARD domain of the NLRP1 directly recruits caspase-1 through CARD–CARD interaction without ASC [8, 9]. However, human NLRP1 also recruits ASC through the PYD domain [10]. For NLRC4, caspase-1 can be recruited to NLRC4 with (through CARD–CARD interaction between NLRC4 and ASC [11]) or without (through CARD–CARD interaction between NLRC4 and caspase-1 [12]) ASC, although differences in size and duration of activated inflammasomes have been observed between these two kinds of NLRC4 inflammasomes [13]. The recruited caspase-1 (also known as caspase-11 in mice) dimerizes and autoclaves to generate p33/p10 species with full protease activity [13]. The cleaved caspase-1 is able to process pro-IL-1β at D26 and D116 and pro-IL-18 at D36 to produce active IL-1β and IL-18 [14]. GSDMD is also cleaved by the caspase-1 to release the amino-terminal domain of GSDMD, which inserts into the plasma membrane to form GSDMD pores leading to pyroptosis [15–17]. The GSDMD oligomerization relies on mitochondria reactive oxygen species (mtROS) provoked by the Ragulator-Rag complex and its downstream mTORC1 [18]. In some cases, cleaved GSDMD can insert into mitochondrial membranes [19, 20]. The leakage of mtROS switches pyroptosis into necroptosis [19]. Through GSDMD pores, are mature IL-1β and IL-18 released into the extracellular environment [21, 22]. A good question is how the GSDMD pores distinguish mature IL-1β and IL-18 from their precursors. A recently published cryo-electron microscopy analysis shows a predominantly negatively charged conduit of the GSDMD pore that favors the passage of mature IL-1β and IL-18 and sequestrates negatively charged IL-1 precursors containing acidic domain [23].

The activation of non-canonical inflammasomes is dependent on caspase-4, caspase-5, caspase-8, and caspase-11 [24]. This pathway depends on caspase-11 in mice and two homologues, caspase-4/5, in humans. This pathway supervises cytosolic Gram-negative bacteria through the detection of lipopolysaccharide (LPS). Caspase-4/5/11 are able to directly bind LPS via CARD domains [25, 26]. Upon sensing LPS, caspase-11 monomers dimerize and acquire the ability to auto-cleave, which generates active caspase-11 species, p32/p10 [27]. For caspase-4/5, LPS is supposed to induce caspase-4/5 oligomerization and activation [28]; however, another research reports that LPS triggers a rapid process of caspase-5, instead of caspase-4, producing active specie of caspase-5 p20 [26]. Counterintuitively, knockdown of caspase-4 does diminish the production of IL-1β, which indicates the involvement of caspase-4 with other signals [26]. Even so, caspase-4, caspase-5, and caspase-11 are able to cleave GSDMD causing the formation of GSDMD pores [16, 17, 29, 30]. These caspases may not directly process pro-IL-1β. Instead, they activate the maturation of pro-IL-1β by NLRP3/ASC/caspase-1 inflammasome via inducing potassium efflux through GSDMD pores [27, 29, 31]. Interestingly, caspase-4 and caspase-11 are able to directly cleave pro-IL-18 in the context of enteric pathogens infection, *Shigella flexneri* and *Salmonella enterica* [32, 33]. In addition, caspase-5 and caspase-11 have been shown to be responsible for the cleavage of pro-IL-1α at D103 in senescent humans and mice [34]. Another non-canonical inflammasome is caspase-8, which is previously known to initiate various cell death cascades, such as apoptosis, anoikis, necroptosis, autophagy, as well as pyroptosis [35]. In macrophages exposed to TLR ligands combined with Fas ligand, caspase-8 has been found to mediate the maturation of IL-1β and IL-18 independently of ASC and caspase-1 [36]. Additionally, dendritic cell-associated C-type lectin-1 (dectin-1) induces activation of caspase-8 and downstream maturation of IL-1β in dendritic cells (DCs) stimulated by fungal and mycobacteria [37]. A similar phenomenon has also been found in macrophages [38]. There seems to be coordination [38] and cross talk between non-canonical caspase-8 inflammasomes and canonical inflammasomes through ASC [39] and NLRP3 [40]. Canonical inflammasomes containing ASC recruit caspase-8 that contributes to the maturation of IL-1β independently of caspase-1 [41]. Non-canonical inflammasomes serve as compensatory mechanisms for canonical inflammasomes, and some cross-talks exist between the two mechanisms. It will be an interesting topic to find how much the stimulators of both inflammasomes overlap.

For termination of the activated inflammasomes, the second self-cleavage of caspase-1 generates p20/p10 species to inactivate caspase-1 [13]. The duration between two self-cleavages of caspase-1 varies in different types of inflammasomes and cells. Macrophages show large ASC inflammasomes and short-term caspase-1 activity, while neutrophils have small ASC inflammasomes and prolonged caspase-1 activity [13]. The fates of the activated cells are dependent on the number of GSDMD pores and the rate of membrane repair known as endosomal sorting complex required for transport (ESCRT) machinery elicited by calcium influx via the GSDMD pores [42]. If inflammasomes are moderately activated to generate limited GSDMD pores, the cells can remain viable through ESCRT machinery by removing GSDMD pores in ectosomes [42]. However, when GSDMD pores are robustly formed in response to serious threats resulting in overwhelming the repair mechanism, the cells would undergo a lytic form of cell death [42], pyroptosis, featured with osmotic swelling and cell rupture [16, 43]. Another possible mechanism that terminates inflammasome activation is autophagy. The NLRP3- and pyrin-mediated inflammasomes are found to localize at the microtubule-organizing center [44]. This group of inflammasomes is then engulfed by double-membrane bilayers and subjected to autophagy degradation, which might be an additional mechanism for inflammasome termination [44]. In summary, the mechanisms of canonical inflammasomes are quite intensively illustrated. It seems compelling to quantify or semi-quantify the inflammasome activation so that more details about the regulation of inflammasome activation can be deciphered.

## Activation mechanisms of the inflammasome sensors

### NLRP1

NLRP1 is the first identified NLR family member with the ability to form inflammasomes [45]. In humans, one *NLRP1* gene exists, while three paralogous *Nlrp1* genes, *Nlrp1a, Nlrp1b,* and* Nlrp1c,* have been found in mice. Human NLRP1 is composed of a pyrin domain, a NACHT domain, a leucine-rich repeat (LRR) domain, a “function to find” domain, and a caspase activation and recruitment domain from N-terminal to C-terminal, while mouse NLRPs lack pyrin domain [46]. Except *Nlrp1c*, *Nlrp1a* [47] and *Nlrp1b* [48] are able to form inflammasomes. At present, the known activators of murine NLRP1 are *Bacillus anthracis* lethal toxin (LeTx) [48], Talabostat (also known as Val-boroPro or PT-100), and 1G244 [49]. Human NLRP1 is a sensor of the double-stranded RNA generated during the replication of the Semliki Forest virus [50]. The LRR domain binds double-stranded RNA enabling the NACHT domain to gain ATPase activity [50]. Additional activators of human NLRP1 include ultraviolet B and ribotoxic stress response [51]. Talabostat and 1G244 [49] also activate human NLRP1, while the target of these two chemicals, dipeptidyl peptidases (DDP)8/9 [49], inhibit human NLRP1. The autoproteolysis of “function to find” domain of NLRP1 releases a C-terminal fragment of NLRP1 [52]. In the resting state, DDP9, full-length NLRP1, and C-terminal fragment of NLRP1 form an inactive trimer that can be disrupted by Talabostat [52]. After stimulation, the liberated C-terminal fragment containing CARD recruits and activates caspase-1 [53, 54]. A similar inactive trimer has been reported in rat NLRP1, which releases a C-terminal fragment with the help of pathogen-induced proteasomal degradation [55]. Although NLRP1 is able to directly recruit caspase-1, ASC is needed for stabilizing the interaction between NLRP1 and caspase-1 [9, 56]. NLRP1 has been reported to be responsible for innate immunity against *Toxoplasma gondii* infection [57].

Homozygous gain-of-function mutation in *NLRP1* gene causes elevated serum IL-1β baseline and juvenile-onset recurrent respiratory papillomatosis [58]. Similarly, *NLRP1* gain-of-function mutations are also associated with multiple self-healing palmoplantar carcinoma and familial keratosis lichenoides chronica through spontaneous inflammasome activation [59]. In addition to tumors, coding polymorphism in NLRP1 increases the risk for autoimmune diseases [60]. The detailed mechanisms linking *NLRP1* mutation and these diseases need further investigation.

### NLRP3

NLRP3 is the most intensively studied NLR family member with broad roles in inflammation and immunity. It possesses a prototypical structure of NLR proteins that contains an N-terminal pyrin domain, a central NACHT domain, and a C-terminal LRR motif. Hence, NLRP3 initiates classical recruitment and activation of ASC through PYD–PYD interaction. The NACHT domain is able to bind and hydrolyze ATP and dATP, which is an essential prerequisite for NLRP3 activation [61]. Structural research finds that mouse NLRP3 forms a membrane-bound and 12–16 mer double-ring cage structure through LRR–LRR and PTD–PYD interactions in a resting state preventing shielded PYDs from nucleating ASC [62]. A similar structure was reported in human NLRP3 that formed 10 mer cages by LRR–LRR interaction in the resting state [63].

Two signals are required for canonical NLRP3 activation, priming signal and activation signal. The priming signal is elicited by PRRs, especially TLRs, and downstream nuclear factor-kappa B (NF-κB) [64, 65]. Once activated, NF-κB promotes NLRP3 and pro-IL-1β expression [64]. Additionally, tumor necrosis factor-α (TNF-α) can also induce pro-IL-1β production [66] and sensitize macrophages to caspase-1 stimulators, such as ATP and silica, in a TNF receptor I- and II-dependent manner [65]. Priming signal also modulates posttranslational modification of NLRP3. TLR4 signal elicits downstream c-Jun N-terminal kinase-1 (JNK-1)-mediated NLRP3 phosphorylation that is essential for inflammasome activation [67]. TLR4 also deubiquitinates NLRP3 through a mechanism involving myeloid differentiation factor 88 (MyD88) and mtROS [68]. In LPS-primed peritoneal macrophages, BRCA1/BRCA2-containing complex 3 (BRCC3) mediates the deubiquitination of NLRP3 thus facilitating NLRP3 activation [69]. The activation signal can be elicited by various stimulators, including DAMPs (such as extracellular ATP, uric acid, and amyloid β fibrils), crystalline particles (such as alum, silica, and asbestos), nigericin, and microbial pore-forming toxins [70–73]. The diverse stimulators activate NLRP3 indirectly through several common pathways such as potassium efflux [73], ROS production [74], lysosomal rupture [75], calcium mobilization [76], mitochondrial DAMPs release [77], and recruitment of NLRP3 to mitochondrial [78], among which potassium efflux has been revealed to be the convergence point. Inflammasome activation by the above mechanisms can be suppressed via potassium efflux blockage [73]. When NLRP3 detects diverse stimuli, acetylation and activation of NLRP3 by lysine acetyltransferase 5 (KAT5) is required for downstream inflammasome assembly with ASC and NIMA-related kinase 7 (NEK7) [79]. NEK7 binds with the LRR domain of NLRP3, which might break the inactive cage of NLRP3 [80]. ATP binds with the NACHT domain causing rotation of WHD–HD2–LRR by approximately 85.4° along the axis between HD1 and WHD resulting in the transformation of the inactivated cage-like NLRP3 into the activated disklike NLRP3 [80]. The PYD domain forms the PYD filament to recruit ASC in the center of the NLRP3 disc [80].

Mutations in *NLRP3* are correlated with cryopyrin-associated periodic syndrome (CAPS) disease spectrum characterized by excessive inflammasome activation in response to harmless stimulators [81, 82]. Elevated IL-1β and IL-18 may drive pathology in different stages of the disease [83]. A possible mechanism of the spontaneous inflammasome activation might be that mutated NLRP3 shows a decreased binding ability with its endogenous inhibitor, cAMP [84].

### NLRC4

NLRC4 is composed of a CARD, a NACHT, and a LRR from N-terminal to C-terminal [85]. Although NLRC4 can directly interact with pro-caspase-1 through CARD–CARD interaction [12, 86], ASC is required for caspase-1 activation and cleavage of pro-IL-1β and pro-IL-18 [86, 87]. On the contrary, direct NLRC4-caspase-1 interaction leads to NLRC4-dependent cell death without efficient cytokine production [86]. ASC in NLRC4 inflammasomes also recruits and activates caspase-8, an apoptotic caspase, that initiates GSDMD-independent cell death when caspase-1 or GSDMD is inhibited [88].

PAMPs from intracellular bacteria are able to elicit NLRC4 inflammasomes [70]. Bacterial flagellin, type III secretion system (T3SS), and type IV secretion system (T4SS) physically bind with NAIPs, which initiates downstream activation of NLRC4 inflammasomes [89–92]. In mice, NAIPs detect multiple components of pathogens, such as NAIP1/2 for T3SS, NAIP2 for bacterial PrgJ, and NAIP5/6 for flagellin [91–93]. Only one type of NAIP-detecting T3SS is reported to exist in humans [92]. However, a different *Naip* transcript variant produces a unique NAIP isoform that detects flagellin [94]. Mutations in NLRC4 cause constitutive IL-1 family cytokine production and macrophage pyroptosis, which is correlated with autoinflammation such as macrophage activation syndrome, neonatal-onset enterocolitis, and lethal periodic fever syndrome [95, 96].

### AIM2

AIM2 does not belong to the NLR family, but it possesses a pyrin domain enabling ASC recruitment [97]. AIM2 has hematopoietic interferon-inducible nuclear antigens with a 200 amino acid repeat (HIN200) domain responsible for detecting double-stranded DNA fragments derived from host’s nuclear genome, mitochondrial genome, virus, and bacteria [97, 98]. Recent work has revealed more complicated downstream events of AIM2 activation, which initiates the assembly of a multi-protein complex containing Pyrin, ASC, caspase-1, and caspase-8 in the context of herpes simplex virus 1 or *Francisella novicida* infection [99]. This multi-protein complex causes PANoptosis instead of pyroptosis that is activated by the canonical AIM2-ASC-caspase-1 pathway [99]. Pathological processes including a variety of infections, autoimmunity, irradiation-induced hematopoietic failure, and gastrointestinal syndrome are associated with AIM2 inflammasome [100–102]. In colorectal cancer, a high frequency of missense and frameshift mutation in *AIM2* has been detected [103]. Lack of AIM2 is associated with increased mortality in colorectal cancer patients and promoted colorectal tumorigenesis in *Aim2*-deficient mice [104, 105].

### Pyrin

Human pyrin protein consists of B30.2 domain, coil-coiled domain, two B-box domains, and pyrin domain from C-terminus to N-terminus, while mouse pyrin does not have the B30.2 domain [106]. RhoA GTPase activates protein kinase N1 (PKN1) and protein kinase N2 (PKN2) causing phosphorylation of pyrin that binds 14-3-3 and is not able to initiate inflammasome in inactivated macrophages [107]. Bacterial toxins such as Clostridium TcdA/B and C3 toxin inhibit RhoA GTPase resulting in dephosphorylation and release of pyrin allowing for downstream ASC- and caspase-1-dependent inflammasome activation [107, 108]. Mutated pyrin is associated with familial Mediterranean fever characterized by decreased binding between pyrin and 14-3-3 or PKN proteins [107].

## Downstream signals of IL-1Rs

IL-1 family members are the common downstream molecules of inflammasomes with diverse downstream functions. IL-1 family is composed of IL-1α, IL-1β, IL-18, IL-33, IL-36Ra, IL-36α/β/γ, IL-37, and IL-38 [109]. Inflammasome-activated caspases are able to mediate maturation of IL-1α, IL-1β, IL-18, and IL-37, whose receptors are IL-1R1/IL-1R3, IL-1R1/IL-1R3, IL-1R5/IL-1R7, and IL-1R5/IL-1R8, respectively [21]. IL-1α is broadly expressed in epithelial cells, endothelial cells, hepatocytes, and fibroblasts, while IL-1β is mainly expressed in myeloid cells. IL-18 is expressed in myeloid cells and epithelial cells [21]. IL-37 is expressed in monocytes, macrophages, lymphocytes, and epithelial cells [110]. IL-1 regulates innate and adaptive immune cells during infection and autoimmune disorders, including fever, angiogenesis, vasodilation, hematopoiesis, leukocyte recruitment, lymphocyte activation, and antibody production [109]. Generally, IL-1α and IL-1β are pro-inflammatory, IL-37 is anti-inflammatory, and IL-18 is pro- or anti-inflammatory depending on the context. IL-1α acts as an alarmin from dying cells and an initiator of an early phase of inflammation, such as the infiltration of neutrophils [111]. Interestingly, pro-IL-1α may enter the nucleus to augment the transcription of pro-inflammatory genes [112]. IL-1β is secreted in response to infection in order to facilitate the recruitment and retention of macrophages [21, 111]. IL-18 promotes leukocyte trafficking, chemokine secretion, nitric oxide production, and adaptive immunity [113]. Additionally, the combination of IL-18 and IL-12 would activate T helper (Th) cells and natural killer (NK) cells causing antiviral and anti-tumor immunity [114]. However, decreased IL-18 production from colonic epithelia is related to more severe colitis indicating the potential anti-inflammatory effect of IL-18 [115]. IL-37 suppresses innate immunity through the downregulation of inflammatory mediators [110]. Both pro- and anti-inflammatory cytokines are cleaved by inflammasomes implying the existence of a mechanism that limits excessive inflammatory response. Compelling findings can be made to dissect when, where, and how the different IL-1 family members are produced in infection, autoimmune disorders, and tumors.

## Inflammasomes in tumor microenvironment (TME)

Progressions of many malignant tumors are regulated by inflammasomes. Present compelling results have revealed the dual role of inflammasomes in TME where inflammasomes promote or inhibit tumor progression depending on different inflammasomes in different tumors. Inflammasomes are involved in tumorigenesis, invasion, metastasis, immune evasion, chemotherapy, and radiotherapy of malignant tumors [116]. It is worthy of note that inflammasomes can be activated in the diverse subgroups of cells in TME, including tumor cells, tumor-associated macrophages, tumor-associated fibroblast, and marrow-derived suppressive cells [117–120]. Additionally, inflammasomes can be activated in distinct conditions resulting in disparate downstream changes. Interestingly, one substance may initiate disparate even opposite mechanisms that regulate inflammasome activation. For example, lactate activates NLRP3 inflammasomes in macrophages by increasing the level of ROS [121]. Meanwhile, lactate also promotes TGF-β production from tumor cells, which induces autophagy in macrophages in small mothers in a decapentaplegic (SMAD)-dependent manner, resulting in ROS clearance and inflammasome attenuation [121].

Novel research approaches such as bioinformatics may contribute to getting a comprehensive landscape of the expression and function of inflammasomes and building links between inflammasomes and clinical data. For example, a pan-cancer analysis has demonstrated that expression levels of NLRP3 can be elevated or dampened in tumor tissues depending on the type of tumors [122]. This analysis also reveals the relationship linking NLRP3 expression with the survival of melanoma and hepatocellular carcinoma, the prognosis of melanoma, and the immunotherapy response, in which elevated NLRP3 expression indicates better survival, improved prognosis, and higher immunotherapy response rate [122]. Another research has established a risk score of inflammasome-related genes in order to predict clinicopathologic characteristics, prognosis, and immune response patterns of kidney renal clear cell carcinoma [123]. More similar research works are needed to understand the role of inflammasomes in the behaviors of tumors.

### Inflammasomes in tumorigenesis

The link between inflammation and cancer has been noticed since Rudolf Virchow’s work in the nineteenth century. Chronic inflammation is critical in multiple stages of tumor progression including tumorigenesis [124]. Tumorigenesis can be fostered by promoting cell survival, augmenting proliferation, or attenuating cell death. As mentioned above, gain-of-function mutations in NLRP1 are associated with multiple self-healing palmoplantar carcinoma [59]. Similarly, people with *NLRP1* variant rs12150220 or *NLRP3* variant rs35829419 are more susceptible to nodular melanoma [125]. The *NLRP3* variants rs10754558 and rs4612666 are significantly associated with gastric cancer [126]. The amino acid mutation Q705K of NLRP3 is associated with pancreatic cancer [127]. Lymphoma susceptibility is also associated with IL-18 (rs1946518) putatively through promoting proliferation and inhibiting apoptosis via unbalance of v-myc myelocytomatosis viral oncogene homolog (c-myc)/tumor protein p53 (TP53) and B-cell lymphoma-2 (Bcl-2)/Bcl-2-associated X protein (Bax) [128]. Whether the pro-tumorigenesis of these inflammasome mutations is underpinned by chronic inflammation remains further investigation. Transgenic mice with the corresponding mutations might help dissect the underlying mechanisms. The pro- and anti-tumorigenesis functions of inflammasomes in different research works are summarized in Table 1.

**Table 1:** Role of inflammasomes in tumorigenesis

Inflammasome compartments	Pro- or anti-tumorigenesis	Type of tumors	Outcomes	References
NLRP1	Pro-tumorigenesis (mutation)	Palmoplantar carcinoma	Gain-of-function mutations in NLRP1 are associated with multiple self-healing palmoplantar carcinoma	[59]
	Pro-tumorigenesis (mutation)	Melanoma	*NLRP1* variant rs12150220 is associated with higher risk of melanoma	[125]
NLRP3	Pro-tumorigenesis (mutation)	Melanoma	NLRP3 variant rs35829419 is associated with higher risk of melanoma	[125]
	Pro-tumorigenesis (mutation)	Gastric cancer	NLRP3 variants rs10754558 and rs4612666 are significantly associated with gastric cancer	[126]
	Pro-tumorigenesis (mutation)	Pancreatic cancer	NLRP3 mutation Q705K is associated with pancreatic cancer	[127]
	Pro-tumorigenesis	Sarcoma	NLRP3 knockout protects mice from methylcholanthrene-induced sarcoma	[132]
	Pro-tumorigenesis	Papilloma	NLRP3-deficient mice are resistant to carcinogenesis-induced papilloma	[296]
	Pro-tumorigenesis	Squamous cell carcinoma	NLRP3 knockout protects mice from 4-NQO-induced squamous cell carcinoma	[133]
	Anti-tumorigenesis	Liver cancer	NLRP3 is downregulated during liver cancer development	[134]
	Anti-tumorigenesis	Colon cancer	NLRP3-deficient mice are susceptible to colitis-associated cancer	[137]
	Anti-tumorigenesis	Colon cancer	NLRP3 knockout increases colitis and colitis-associated cancer	[136]
NLRC4	Anti-tumorigenesis	Colon cancer	NLRC4-deficient mice show enhanced tumor formation	[141]
Pyrin	Anti-tumorigenesis	Colon cancer	Pyrin knockout increases colitis and tumorigenesis through promoting intestinal barrier integrity	[138]
ASC	Pro-tumorigenesis	Gastric cancer	ASC knockout suppresses spontaneous gastric cancer	[129]
	Pro-tumorigenesis	Cecal cancer	ASC knockout suppresses spontaneous cecal cancer	[130]
	Pro-tumorigenesis	Skin cancer	Conditional knockout of ASC in myeloid cells reduces chemical-induced skin cancer	[143]
	Anti-tumorigenesis	Squamous cell carcinoma	ASC knockout promotes tumorigenesis through decreasing anti-tumor immunity	[135]
	Anti-tumorigenesis	Skin cancer	Conditional knockout of ASC in keratinocytes augments chemical-induced skin cancer	[143]
Caspase-1	Pro-tumorigenesis	Cecal cancer	Caspase-1 inhibitor suppresses spontaneous cecal cancer	[130]
	Pro-tumorigenesis	Squamous cell carcinoma	Caspase-1 knockout protects mice from 4-NQO-induced squamous cell carcinoma	[133]
	Anti-tumorigenesis	Squamous cell carcinoma	Caspase-1 knockout promotes tumorigenesis through decreasing anti-tumor immunity	[135]
	Anti-tumorigenesis	Colon cancer	Caspase-1-deficient mice show enhanced tumor formation	[141]
Caspase-11	Anti-tumorigenesis	Colon cancer	Caspase-11 knockout mice are more susceptible to colitis-associated cancer	[139]
IL-1α	Anti-tumorigenesis	Breast cancer	IL-1α knockout mice show higher tumor burden and elevated death rate	[140]
IL-1β	Pro-tumorigenesis	Gastric cancer	Overexpression of IL-1β causes spontaneous gastric cancer	[131]
	Pro-tumorigenesis	Lung cancer	Anti-inflammatory therapy by canakinumab reduced lung cancer incidence	[284]
IL-18	Pro-tumorigenesis (mutation)	Lymphoma	*IL-18* variant rs1946518 is associated with higher risk of lymphoma	[128]
IL-1R1	Anti-tumorigenesis	Breast cancer	IL-1R1 knockout mice show higher tumor burden and elevated death rate	[140]

Plenty of spontaneous tumor models and stimulator-induced tumor models have revealed the relationship between inflammasome pathway and tumorigenesis. In some cases, suppression of inflammasomes attenuates tumorigenesis. For example, ASC knockout suppresses tumorigenesis in glycoprotein 130 (*gp130)*^*F/F*^ mice that develop spontaneous intestinal-type gastric cancer [129]. ASC ablation reduces mature IL-18 from gastric tumor epithelium causing augmented caspase-8-like apoptosis. Interestingly, this mechanism does not involve canonical IL-1β maturation and inflammation elicited by IL-1β [129]. Similarly, ASC knockout, caspase-1 inhibition, or removing germ reduces spontaneous cecal carcinogenesis in *AhR*^*−/−*^ mice, indicating bacteria-triggered inflammation and inflammasomes to be detrimental factors during tumorigenesis [130]. The pro-tumorigenesis effect of microbe could be partially attributed to stimulated inflammasomes and downstream IL-1β/NF-κB/IL-6/signal transducer and activator of transcription 3 (STAT3) pathway [130]. Consistent with these findings, overexpression of IL-1β in the stomach of mice leads to spontaneous gastric inflammation and cancer [131]. Recruitment and activation of myeloid-derived suppressor cells (MDSCs) by IL-1β through IL-1R1/NF-κB are the links between IL-1β and tumorigenesis [131]. For chemically induced models, knockout of NLRP3 protects mice from methylcholanthrene-induced sarcoma in NK cells and interferon gamma (IFN-γ)-dependent manner [132]. Similarly, *Nlrp3*^*−/−*^ mice and *Caspase-1*^*−/−*^ mice show less and later tumor incidence when challenged with the carcinogen, 4-nitroquinoline 1-oxide (4-NQO) [133].

On the contrary, several other findings have suggested inflammasome pathway to function as a protector during tumorigenesis. Downregulation of several NLRP3 inflammasome components has been demonstrated in multistage hepatocarcinogenesis [134]. For chemically induced squamous cell carcinoma, the protective roles of ASC and caspase-1 through recruiting immune cells during tumorigenesis have been proven [135]. In colitis-associated cancer models, mice lacking ASC, caspase-1, or NLRP3 show more severe colitis and accentuated tumorigenesis [136]. Similarly, NLRP3-deficient mice are susceptible to colitis-associated cancer [137]. The attenuated hematopoietic cell-derived IL-1β and IL-18 at the tumor site of *Nlrp3*^*−/−*^ mice are found to be the key for inflammation and tumorigenesis [136]. These findings are coincident with results from pyrin knockout mice that also develop more severe colitis and larger tumor burden [138]. The effect of IL-18 is further verified by the administration of rIL-18 that reduces inflammation and tumorigenesis [138]. Similarly, *caspase-11*^*−/−*^ mice are more susceptible to colitis-associated cancer compared with wild-type littermates [139]. Besides deficient IL-18 production, impaired IL-1β is also responsible for tumorigenesis [139]. IL-1β produced by caspase-11-associated inflammasomes is able to conversely induce expression of caspase-11 that stimulated STAT-1 leading to inhibited tumorigenesis [139]. The effector cytokines of inflammasomes can be different when it comes to spontaneous breast cancer mice models where genetic blockage of IL-1α/IL-1R1 signal develops higher tumor burden and increased mortality rate [140] implying similar roles of IL-1α, IL-1β, and IL-18 in tumorigenesis. Additionally, inhibiting inflammasomes by caspase-1 knockout also mediates tumorigenesis by suppressing caspase-1-mediated cell death. Caspase-1-deficient or NLRC4-deficient mice show increased colonic epithelial cell proliferation and reduced tumor cell apoptosis resulting in enhanced tumor formation in the colitis-associated colorectal cancer models [141].

It is worthy of note that the regulation of tumorigenesis by inflammasomes may change during the development of malignant tumors. Upregulation of NLRP3 inflammasome components has been detected in tissues of hepatitis and cirrhosis, while the expression levels are diminished in hepatocellular carcinoma [134]. Knockdown of ASC shows opposite effects on the tumorigenesis of metastatic and primary melanoma cells. Silencing ASC with short hairpin RNA suppresses tumorigenesis in metastatic melanoma, while it enhances tumorigenesis in primary melanoma [142]. This contrary phenotype can be explained by different downstream NF-κB activity, which is inhibited in primary melanoma yet augmented in metastasis melanoma by ASC [142]. Additionally, the role of inflammasome components in tumorigenesis may change depending on where they are expressed. Conditional knockout of ASC in myeloid cells reduces chemical-induced skin cancer, while ASC-specific deletion in keratinocytes augments tumorigenesis [143]. Thus the relationship between inflammasomes and tumorigenesis seems to be dependent on stages of disease and cell types in the microenvironment.

Besides IL-1 family members, tumor growth can also be regulated by GSDMD, whose elevation is associated with more advanced TNM stages in non-small cell lung cancer (NSCLC) patients. Knockdown of GSDMD inhibits tumor growth through promoting the mitochondrial apoptotic pathway and inhibiting epidermal growth factor receptor (EGFR)/AKT signaling [144]. On the contrary, GSDMD is downregulated in gastric cancer cell lines and tissues, in which diminished GSDMD expression levels lead to promoted tumor cell proliferation through accelerating S/G2 cell transition [145]. GSDMD expression is negatively associated with the activation of STAT3, extracellular signal-regulated kinase (ERK), and phosphatidylinositol 3-kinase (PI3K)/AKT signal [145]. Different downstream signals of GSDMD in disparate tumors may explain these controversial findings.

### Inflammasomes in tumor cell death

Pyroptosis mediated by the formation of GSDMD pores is the downstream event of Inflammasomes. Thus mediation of tumor cell death by inflammasomes is mainly achieved by GSDMD-induced pyroptosis. Notably, GSDMD-mediated pyroptosis includes not only non-canonical/canonical inflammasome-dependent pyroptosis but also apoptotic caspases-8-mediated pyroptosis [146]. Here we focus on the non-canonical/canonical inflammasome-dependent pyroptosis. Despite the unexpected findings from NSCLC that higher GSDMD expression is correlated with advanced TNM stages and poor prognosis and that GSDMD knockdown induces apoptosis of tumor cells [144], the majority of the findings imply that downregulated GSDMD suppresses pyroptosis and that activating GSDMD boosts pyroptosis.

In gastric cancer, downregulated GSDMD promotes tumor growth [145]. The GSDMD-mediated pyroptosis might happen during conventional anti-tumor therapy. For example, cisplatin has been demonstrated to be involved in NLRP3/caspase-1/GSDMD pyroptosis pathway in breast cancer cells [147]. Indeed, many researchers have found a host of chemicals that induce GSDMD-dependent pyroptosis of tumor cells through various mechanisms. For example, metformin leads to GSDMD-mediated pyroptosis in chemo-refractory esophageal squamous cell carcinoma [148]. Anthocyanin activates pyroptosis in oral squamous cell carcinoma cells via enhancing the expression of NLRP3, caspase-1, and IL-1β [149]. Similarly, 4-hydroxybenzoic acid selectively induces pyroptosis in lung cancer cell line A549 through activating transcription of caspase-1, IL-1β, and IL-18, while normal lung epithelial cells are not affected [150]. Simvastatin also induces pyroptosis in A549 and H1299 via provoking NLRP3 pathway [151]. Val-boroPro, a DPP8/9 inhibitor, evokes caspase-1-dependent pyroptosis in human acute myeloid leukemia [152]. Docosahexaenoic acid triggers caspase-1 activation, GSDMD maturation, and IL-1β secretion in breast cancer cell line, MDA-MB-231, through lysosomal damage and ROS formation [153]. Lysosomal rupture seems to be the common downstream event of different interventions causing pyroptosis in cancer cells [153–155]. Non-canonical inflammasome signal, GSDMD/caspase-4, elicited by 2-(anaphthoyl) ethyltrimethylammonium iodide contributes to the pyroptosis of epithelial ovarian cancer cells [156]. LPS is also able to evoke non-canonical inflammasome caspase-11-mediated pyroptosis in lung cancer cells [157]. Besides the great number of chemicals, various delicate nanoparticles have been developed to foment inflammasome-mediated pyroptosis [155, 158].

A possible explanation of the conflicting findings in NSCLC clinical data and others could be the different focuses of these research works. In most cases, various chemicals initiate GSDMD-mediated pyroptosis in different cancer cells; however, few of these research works focus on the downstream events of pyroptosis. For example, IL-1β produced from pancreatic cancer cells treated with LPS plus ATP increases cell proliferation, indicating pyroptosis in cancer cells to be a two-edged sword [159]. In another word, pyroptosis of tumor cells may start a set of downstream changes that promote tumor progression, which will be discussed in the following parts.

### Inflammasomes in angiogenesis of tumors

In tumor tissues, the angiogenesis-derived blood vessels are disorganized, immature, and permeable [160], which are required for many malignant behaviors including tumor metastasis and tumor growth [161]. The involvement of inflammasome signals in angiogenesis requires vascular endothelial growth factor (VEGF), hypoxia-inducible factor-1$\mathrm{\alpha }$ (HIF-1$\mathrm{\alpha }$), and C-X-C motif chemokine ligand 2 (CXCL2) [162, 163]. Overexpression of IL-1$\upbeta$ in lung cancer cells is the cause of obviously elevated VEGF and CXCL2 secretion, which facilitates angiogenesis and tumor growth [164]. Mechanistically, IL-1$\upbeta$ upregulates HIF-1$\mathrm{\alpha }$ expression in the NF-κB-dependent manner [165]. The HIF-1$\mathrm{\alpha }$ is the direct upstream mediator of VEGF expression [165]. Knockout of either IL-1$\upbeta$ or IL-1$\mathrm{\alpha }$ hampers angiogenesis and tumor growth [166]. Importantly, the inhibitory effects of IL-1$\upbeta$ are more obvious than that of IL-1$\mathrm{\alpha }$ [166]. Except tumor cells, macrophages treated by hypoxia also secrete IL-1$\upbeta$ that enhances angiogenesis by VEGF [167]. Additionally, this pathway has also been reported in adipocytes [168]. The IL-1$\upbeta$ and VEGF interaction is an autoinduction circuit; however, inhibiting IL-1$\upbeta$ has been proven to be a better choice than inhibiting VEFG [169]. In general, IL-1 s, VEGF, and HIF-1$\mathrm{\alpha }$ form a network of angiogenesis. The failure of anti-VEGF might be rescued by the addition of anti-IL-1 s.

### Inflammasomes in invasion and metastasis

Invasion and metastasis are two crucial malignant behaviors of tumors. Degradation of extracellular matrix, angiogenesis, and migration through basal membranes are key steps during invasion and metastasis. Abnormal inflammasome activation participates in the mediation of these steps. Alterations of inflammasome expression in different tumors have been reported. For instance, NSCLC shows overexpressed AIM2, while lung adenocarcinoma and small cell lung cancer (SCLC) show upregulated NLRP3 [170]. Expression of NLRP3 is upregulated in bladder cancer, especially at the early tumor stages [171]. NAIP, the regulator of NLRC4, is also overexpressed in high-risk and high-grade bladder cancer patients [171]. Many research works focus on the relationship between cancer metastasis and inflammasome activation in myeloid cells, because IL-1β in TME is predominantly produced by myeloid cells [172–174]. Additionally, activations of inflammasomes in cancer-associated fibroblasts [117] and tumor cells [175] are also associated with tumor metastasis.

**Fig. 2:**
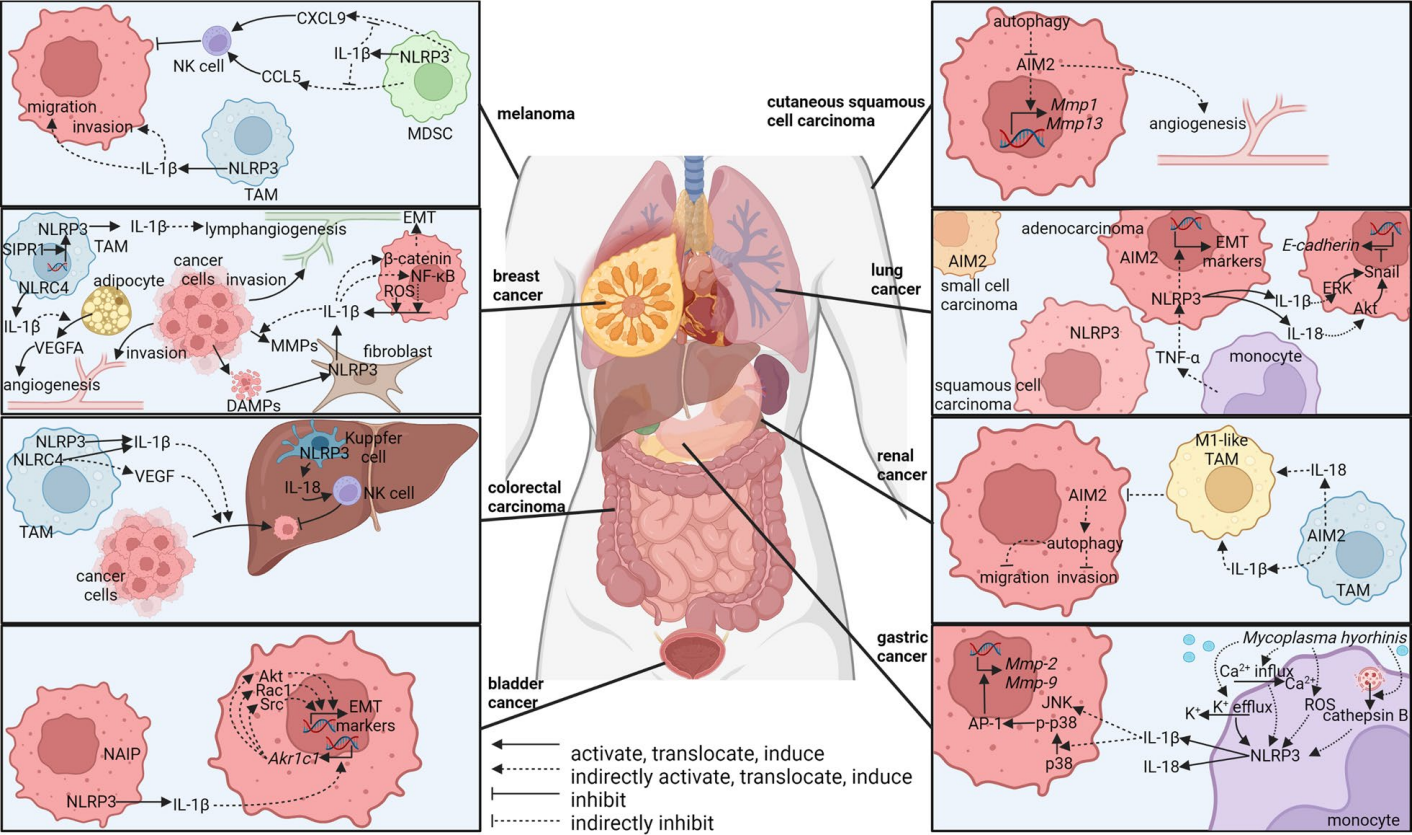
Role of inflammasomes in tumor metastasis

Although IL-1β is regarded as a marker of M1-like macrophages that activates anti-tumor immunity in some cases [176, 177], abnormal inflammasome activation in tumor-associated macrophages (TAMs) has been manifested to be a promoter of invasion and metastasis in many kinds of tumors. Clinical data have shown a positive correlation between the activation of inflammasomes, especially NLRP3, and metastasis, late clinical stages, and poor survival rate in breast cancer and lung cancer patients [118, 178]. Blocking IL-1 signal by anakinra or canakinumab reduces cancer cells in circulation and suppresses metastasis of breast cancer [179]. In balder cancer, IL-1β induces expression of aldo–keto reductase 1C1 (AKR1C1), which is associated with invasion, cisplatin resistance, and metastasis of cancer cells [180]. However, inflammasome activation suppresses tumor cell invasion and metastasis in other cases [181, 182]. The opposite findings indicate a double-edged role of inflammasomes in TME. The involvement of inflammasomes in the metastasis of different tumors is summarized in Fig. 2.

#### NLRP3 in invasion and metastasis of tumors

NLRP3 has been reported to promote the invasion and metastasis of tumor cells in some cases. Many research works focus on myeloid cell-derived NLRP3 signal. In primary colorectal tumors, NLRP3 is overexpressed in TAMs along the tumor boundaries [183]. NLRP3 can be activated through cross-talk between TAMs and cancer cells leading to promoted migration of cancer cells in IL-1β dependent manner [183]. On the contrary, antagonists targeting NLRP3 or caspase-1 suppress the migration of colorectal cancer cells in vitro, and knockout of NLRP3 decreases liver metastasis nodes in vivo [183]. In breast cancer, NLRP3-expressing macrophages are associated with nodal metastasis, distant metastasis, and poor survival rate [118]. S1P receptor 1 (S1PR1) from TAMs promotes NLRP3 expression and IL-1β production, which initiates lymphangiogenesis, a pivotal step of metastasis [118]. In gastric cancer, migration and invasion of cancer cells can be promoted by monocyte-derived IL-1β, which is stimulated by *Mycoplasma hyorhinis* in a TLR2-dependent manner [184]. In response to *Mycoplasma hyorhinis* infection, elicited cathepsin B, K^+^ efflux, Ca^2+^ influx, and ROS production activate NLRP3 inflammasome and IL-1β secretion [184]. IL-1β enhances migration, invasion, and metastasis in gastric cancer cells through elevated expression of MMP-2 and MMP-9 [185]. Mechanistically, IL-1β activates p38, which is an upstream signal of activator protein-1 (AP-1)-dependent transcription of MMPs [185]. IL-1β also elicits JNK in cancer cells [185]. Although JNK has been reported to promote metastasis [186, 187], JNK is not related to migration and invasion here [185]. These findings imply a link between infection-related inflammation and pro-tumor inflammation. In melanoma, macrophage-derived NLRP3/IL-1β pathway promotes migration and invasion of melanoma cells, which can be blocked through NLRP3 knockout, caspase-1 knockout, or NLRP3 inhibitor, celastrol [172]. Similarly, NLRP3 from a subgroup of CD11b^+^ Gr-1^int^ myeloid cells has been shown to foster tumor metastasis of B16-F10 [132]. CD11b^+^ Gr-1^int^ myeloid cells from *Nlrp3*^*−/−*^ mice produce higher levels of C–C motif chemokine ligand 5 (CCL5) and C-X-C motif chemokine ligand 9 (CXCL9) that are responsible for elevated recruitment and activation of NK cells in tumor microenvironment resulting in  a lower number of lung metastasis [132].

Cancer-associated fibroblasts are another subset of cells that sense DAMPs and secrete IL-1β through the inflammasome pathway [117]. The NLRP3-IL-1β pathway from fibroblasts facilitates tumor growth and lung metastasis through intensifying immune suppression, expression of invasive markers in tumor cells, and expression of endothelial cell-derived adhesion molecules [117].

For cancer cell-derived inflammasomes, NLRP3 inflammasomes and downstream IL-1β secretion can be activated by breast cancer susceptibility gene 1 (BRCA1) deficiency through ROS production leading to promoted metastasis in breast cancer cells [188]. Inflammasome inhibitor, glibenclamide, treatment ameliorates *Brca1* mutant breast cancer metastasis [188]. Additionally, inflammasome pathway and IL-1β production can also be elicited by ATP or TNF-α through the P2Y2 receptor (P2Y_2_R) in breast cancer cells, which promotes the expression of matrix metallopeptidase-9 (MMP-9) and resultant invasion [175]. The detailed downstream pathway of IL-1β might include the induced β-catenin accumulation and translocation to the nucleus through AKT/glycogen synthase kinase 3β (GSK3β) signal resulting in upregulation of c-MYC, Cyclin D1 (CCDN1), Snail family transcriptional repressor 1 (SNAIL1), and MMP-2 that promote migration, invasion, and proliferation [189]. Another downstream event of IL-1β in breast cancer is NF-κB that enhanced cell invasion and activation [190]. Interestingly, phosphorylated NF-κB mediates further production of IL-1β. The positive feedback loop between NF-κB activation and IL-1β production can be disturbed by NF-κB inhibitors, zerumbone and Bay11-7085 [190]. In lung adenocarcinoma, higher NLRP3 level in patient specimens is correlated with latter stage and lymph node metastasis [178]. NLRP3 activation is the downstream event of TNF-α that induces epithelial–mesenchymal transition (EMT) in lung adenocarcinoma cell line A549 [178]. LFG-500, an inhibitor of NLRP3 inflammasome, suppressed EMT, migration, and metastasis of A549 [178]. Tumor cell-derived IL-1β and IL-18 elicited by NLRP3 are responsible for EMT through activating ERK and AKT signal resulting in strengthened migration [191].

However, NLRP3-mediated production of IL-1β and IL-18 inhibits the formation of metastatic lesions in other cases. Although IL-1β produced by NLRP3 promotes the migration of colorectal cancer cells [183], Saleh et al. have found that NLRP3 activation in liver macrophages (Kupffer cells) attenuates colorectal cancer metastatic growth [182]. NLRP3 in Kupffer cells mediates IL-18 secretion, which facilitates the maturation and tumoricidal activity of NK cells [182].

In summary, NLRP3 inflammasome signal seems to promote or suppress tumor metastasis depending on different kinds of tumors and tissues. It is possible that diverse patterns of IL-1β and IL-18 production exist in different cell subsets leading to discrepancies in downstream events.

#### NLRC4 in invasion and metastasis of tumors

In the context of non-alcoholic fatty liver disease, the number and size of colorectal cancer liver metastasis nodes are significantly increased through the activation of NLRC4 in TAMs [192]. NLRC4 activity is correlated with M2-like polarization of TAMs, upregulated IL-1β, VEGF expression, and increased vascularity [192]. NLRC4 inflammasomes are also able to mediate the progression of breast cancer in the context of obesity [168]. Activated NLRC4 inflammasomes from tumor-infiltrating myeloid cells produce IL-1β, which promotes vascular endothelial growth factor A *(Vegfa)* expression in adipocytes facilitating angiogenesis, a key step in metastasis [168]. Thus abnormal NLRC4 activation in TME may facilitate tumor metastasis.

#### AIM2 in invasion and metastasis of tumors

Elevated expression of AIM2 has been detected in cutaneous squamous cell carcinoma than in normal skin [193]. AIM2 knockdown results in reduced invasion proteinases, MMP-1 and MMP-13, decreased cell viability, suppressed vascularization, and onset of apoptosis [193]. Partly through activating autophagy that suppresses AIM2, dihydroartemisinin shows an inhibitory effect on cutaneous squamous cell carcinoma [194]. A bioinformatics analysis that establishes a risk-scoring system involving inflammasomes indicates higher AIM2 expression may relate to poorer overall survival in renal carcinoma patients [123].

However, there are also some controversial results. In renal carcinoma patients, low AIM2 expression is correlated with lymph node metastasis, poor 5-year overall survival, and poor disease-specific survival [195]. In renal carcinoma cell lines, 786-O and OSRC-2, tumor cell-derived AIM2 inhibits cell migration and invasion by enhancing autophagy [195]. TAM-derived AIM2 inflammasomes in renal carcinoma also show a protective role in tumor invasion and metastasis through a different mechanism [196]. This protective role is based on increased M1-like polarization and reduced M2-like polarization of TAMs elicited by AIM2 inflammasomes [196]. The inflammasome inhibitor, Ac-YVAD-CMK abrogates M1 polarization, while overexpression of AIM2 in macrophages inhibits tumor growth and metastasis [196]. Whether AIM2 promotes tumor progression through some unknown mechanisms should be further elucidated.

Together, cross-talk between the AIM2 pathway and other pathways might exist, which could explain the double-faced role of AIM2 in different research works.

### Inflammasomes in immune evasion

The phenomenon has been well described that tumors achieve consistent progression through immune evasion. Tumor cells may implement alteration of Fas receptor, upregulation of programmed cell death-ligand 1 (PD-L1), and downregulation of major histocompatibility complex class I (MHC-I) [197, 198]. In TME, M2-like macrophages, MDSCs, and regulatory T cells (Tregs) are recognized as hallmarks of immune-suppressive environment that facilitate immune evasion of tumor cells [197]. Inflammasome components can be expressed and activated by various stimulators in cancer cells [119, 195, 199], fibroblasts [117], and macrophages [196, 200] resulting in the secretion of IL-1β and IL-18, which further modulates the expression of PD-L1 in tumor cells and recruitment of immune-suppressive cells in TME. A feed-forward process may be established when inflammasomes direct pyroptosis that released DAMPs causing further activation of inflammasomes and recruitment of immunosuppressive cells [201]. Besides the DAMP-mediated feed-forward process, the IL-1 signal also forms a feed-forward loop with IL-6. Constitutively activated NLRP3 in melanoma secretes IL-1β that initiates IL-6 secretion through stimulating IL-1R [202]. The IL-6 further binds to IL-6R to stimulate Janus kinase (JAK)/STAT3 cascade allowing for further production of IL-6, which synergizes with IL-1β to activate MDSCs [202]. Similarly, NLRP3 is overexpressed in tissues of head and neck squamous cell carcinoma resulting in increased IL-1β concentration in blood, spleen, draining lymph nodes, and tumor tissues [203]. The immunosuppressive cells, Tregs, MDSCs, and TAMs are positively correlated with NLRP3 inflammasome activation, which can be eradicated by MCC950 [203] or OLT1177 [202], two NLRP3 inhibitors. A similar result has been reported in *Nlrp3*^*−/−*^ mice that demonstrate dramatically better response to dendritic cell vaccination with a fivefold reduction in MDSCs [204]. Besides IL-1β, IL-18 production from multiple myeloma niche has also been reported to be correlated with expanded MDSCs, diminished T cells, and poor overall survival [205]. However, IL-1β and IL-18 are able to promote T cell immunity against cancer in other cases [206, 207]. CD4^+^ T cell-derived or exogenous IL-18 promotes proliferation and anti-tumor activity of CD8^+^ T cells and chimeric antigen receptor (CAR)-T cells [207]. What’s more, knockout of ASC or caspase-1, two downstream components of inflammasomes, leads to an immunosuppressive environment characterized by decreased NK cells, DCs, CD4^+^ T cells, and CD8^+^ T cells and increased Foxp3^+^ T cells [135]. The conflicting findings may be resolved by quantifying the concentration of IL-1β and IL-18 in TME instead of simply describing the changes in their concentration. It is possible that IL-1β and IL-18 can initiate distinct immune patterns in different concentrations. Another good question is where the inflammasomes, IL-1β, and IL-18 are expressed. Understanding the details of inflammasome activation in different cell subsets may help depict the network of inflammation and immune supervision in TME and develop therapeutic interventions.

Tumor cell-derived inflammasome activation creates an immune-suppressive environment in most cases. NLRP3 inflammasomes in melanoma cells can be activated by a combination of agonistic anti-PD-L1 antibody and IFN-γ through PD-L1/STAT3/protein kinase R (PKR) signal axis or direct contact of tumor cells and antigen-specific CD8^+^ T cells at the existence of anti-programmed cell death protein-1 (PD-1) [199]. Activated NLRP3 inflammasomes elicit autocrine heat shock protein 70 (HSP70)/TLR4 signal pathway followed by Wnt family member 5A (Wnt5a)/C-X-C motif chemokine ligand 5 (CXCL5)/C-X-C motif chemokine receptor 2 (CXCR2) signal pathway that recruits granulocytic MDSC to suppress immune supervision of CD8^+^ T cells [199]. Tumor cell-derived IL-1β establishes an immunosuppressive milieu characterized by M2-like macrophages, MDSC, Th17 cells, and CD1d^hi^ CD5^+^ regulatory B cells in pancreatic cancer [119]. Deprivation of IL-1β through shRNA or neutralizing antibody restores anti-tumor immunity and improves the effect of anti-PD-1 therapy [119]. An autoinflammatory loop has been reported in melanoma, where tumor cells produce IL-1β and IL-6 through IL-1β/IL-6/STAT3 axis allowing for the activation of MDSCs [202]. Besides IL-1β, IL-18 secreted by tumor cells through NLRP3 is positively correlated with PD-L1 expression and negatively correlated with cytotoxic T cells [208]. NLRP3 inhibitor, MCC950, ameliorates anti-tumor immunity and dampens xenograft growth [208]. However, another research has found that NSCLC-derived IL-18 stimulates anti-tumor IFN-γ production from a minor part of CD8^+^ T cells (T-bet^+^Eomes^+^) that expresses a high level of IL-18R [209].

Myeloid cell-derived inflammasome activation seems to be beneficial for anti-tumor immunity. For example, NLRP3 inflammasomes in DCs are activated allowing for IL-1β secretion when ATP from dying tumor cells acts on P2 purinergic receptors (P2X7) purinergic receptors from DCs [206]. IL-1β from DCs is the key to the priming of IFN-γ-producing CD8^+^ T cells [206]. Besides IL-1β, myeloid cell-derived IL-18 also facilitates anti-tumor immunity [210]. Inhibiting CD39, an ecto-enzyme converting extracellular ATP to AMP, through antibody activates NLRP3 inflammasomes leading to IL-18 secreting that expands intra-tumor effector CD4^+^ and CD8^+^ T cells [210]. However, NLRP3 expression and NLRP3-mediated secretion of IL-1β and IL-18 from alveolar macrophages in NSCLC and SCLC are attenuated when compared with peripheral blood leukocytes [200]. A possible reason might be the impaired TLR4/LPS pathway in alveolar macrophages from tumor tissues [200], but the details are still fuzzy. Additionally, TAM-derived AIM2 is able to reverse M2-like TAMs into M1-like TAMs that possess anti-tumor activities [196].

Fibroblast-derived IL-1β is secreted through NLRP3 inflammasomes, which are activated by various DAMPs including necrotic fluid from breast cancer cells [117]. The NLRP3/IL-1β pathway is responsible for the recruitment of monocytic MDSCs (CD11b^+^Ly6C^high^Ly6G^−^) or granulocytic MDSCs (CD11b^+^Ly6C^low^Ly6G^+^) depending on the genetic background of mice [117].

Interestingly, the IL-1 signal has distinct effects on different cell subsets in even one colorectal tumor model. IL-1R1 ablation in T cells dampens the production of IL-17 and IL-22 that promotes tumor-elicited inflammation and tumor progression [211]. Similarly, IL-1R1 knockout also alleviates tumorigenesis [211]. However, when IL-1R1 is knockout in neutrophils, bacterial invasion into tumors potentiates inflammation allowing for enhanced tumor progression [211]. This phenomenon could be explained by the diverse background pathways in different cell subsets.

**Fig. 3:**
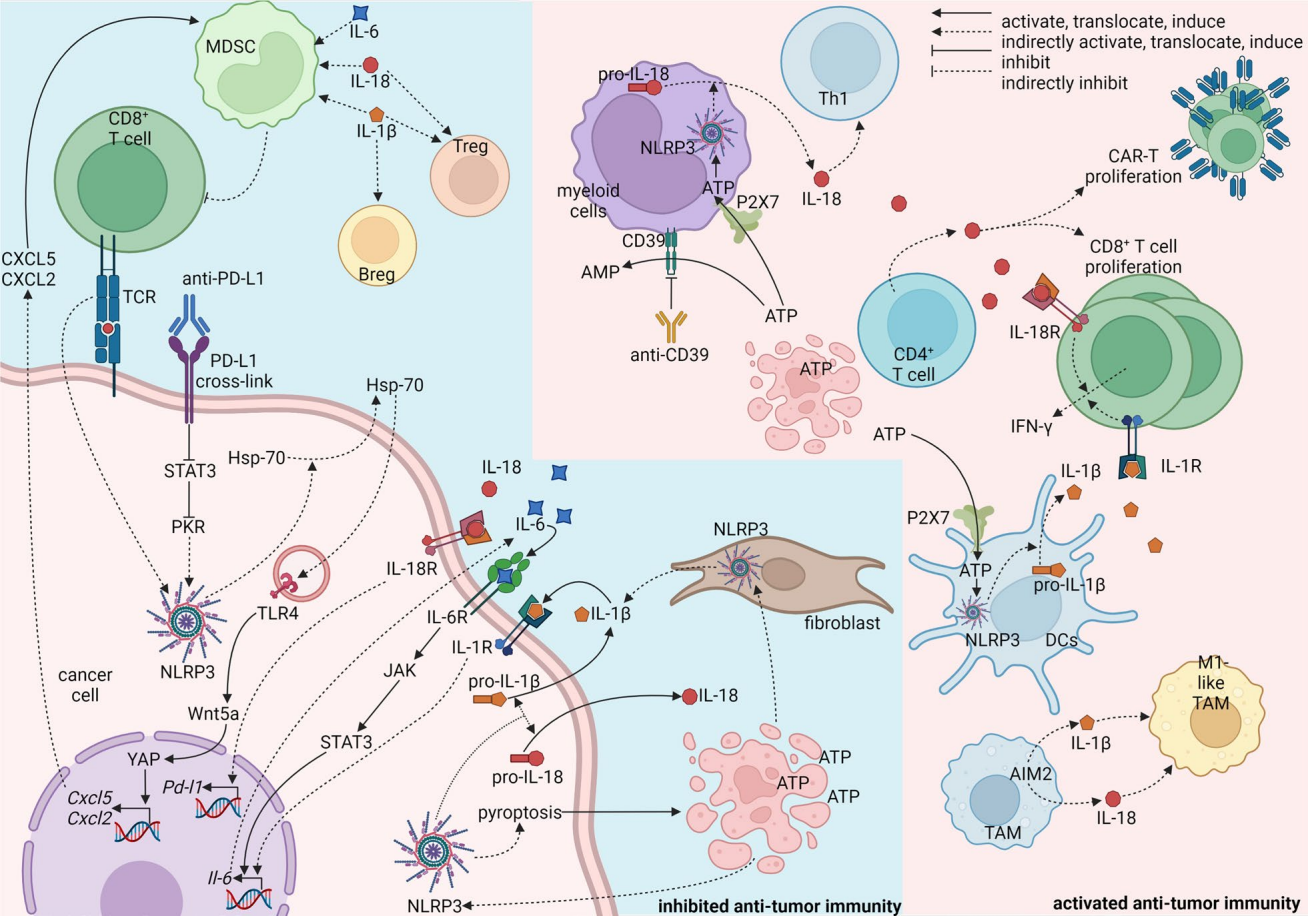
Role of inflammasomes in anti-tumor immunity

The mechanisms of the inhibited/promoted anti-tumor immunity by inflammasomes are summarized in Fig. 3. It seems that tumor-derived and fibroblast-derived inflammasomes allow for immunosuppressive TME, while myeloid cell-derived inflammasomes cause increased anti-tumor immunity. A possible explanation might exist in the different size and duration of inflammasomes between myeloid cells and other cells in TME. These differences have been revealed between macrophages and neutrophils [13] indicating that inflammasomes can be activated to diverse extents causing disparate downstream mechanisms. It would be a tempting work to elucidate the different inflammasome activation and downstream changes in diverse cell subsets. Another possible explanation may be the discrepancy in the concomitant signals between myeloid cells and other cells. For DCs and macrophages, secretion of IL-1β and IL-18 is accompanied by antigen presentation [206] or T cell recruitment [177], while inflammasome activation in tumor cells is accompanied by immunosuppressive signals such as PD-1/PD-L1 [208]. More work is needed for a better understanding of the cross-talk between IL-1 family signals with other signals.

## Inflammasomes in classical therapy

### Inflammasomes in chemotherapy

Chemotherapy is a canonical choice for patients with malignant tumors. In general, chemotherapy agents activate inflammasomes in cancer cells and myeloid cells through several pathways, which may enhance or dampen the anti-tumor effects of the agents. NLRP3 is the most commonly activated inflammasome by agents including doxorubicin [212], daunorubicin [212], melphalan [213], gemcitabine [120], fluorouracil (5-FU) [120], cytarabine [213], methotrexate [213], paclitaxel [214], etoposide [213], vincrisitine [213], and cisplatin [215]. In different situations, the downstream mechanisms of the NLRP3 activation might promote or inhibit the malignant behaviors of tumors including tumor growth, metastasis, and drug resistance.

Direct anti-tumor effects of doxorubicin and cisplatin on malignant mesothelioma rely on pyroptosis attributed to increased NLRP3 expression and caspase-1 activation [215]. NLRP3 is also involved in FL118-mediated pyroptosis, which can be reversed by the NLRP3 inhibitor, MCC950 [216]. Indirectly, mitoxantrone induces anti-tumor immunity against fibrosarcoma, characterized mainly by enhanced CD8^+^ T cell activation, through myeloid cell-derived IL-1β produced by NLRP3 inflammasomes [217]. Mechanistically, the inflammasome activation relies on phosphatase and tensin homolog (PTEN) that directly dephosphorylates NLRP3 to initiate inflammasome assembly [217]. Likewise, myeloid cell-derived IL-1β is associated with anti-tumor immunity in patients [217]. Collectively, inflammasome signal is involved in the anti-tumor effects of chemotherapy agents directly through provoking pyroptosis and indirectly through activating immune cells.

However, IL-1β production elicited by anti-tumor agents from various cell subsets in TME is not always beneficial. MDSC-derived IL-1β induces IL-17 secretion by CD4^+^ T cells leading to curtailed anti-tumor effect of 5-FU against several kinds of tumors including lymphoma, breast cancer, melanoma, and lung cancer [120]. In MDSCs, activation of NLRP3 by 5-FU, as well as gemcitabine, is underpinned by lysosomal permeabilization, which is the causative factor for cathepsin B leakage resulting in the stimulation of NLRP3/caspase-1 signal [120]. Although blocking macrophage-derived IL-1β retards tumor growth during paclitaxel therapy, tumor metastasis and M2-like polarization of TAMs are enhanced indicating IL-1β to be a double-edged sword [214]. Similarly, tumor cell-derived inflammasome may also be detrimental. In patients with oral squamous cell carcinoma, 5-FU application increases expression and activation of NLRP3 that is associated with higher tumor stage, moderate/poor differentiation, and poor prognosis [133]. ROS induced by 5-FU has been revealed to be the causal factor for the expression and activation of NLRP3 and secretion of IL-1β, which further mediates drug resistance [133]. Likewise, gemcitabine-resistant triple-negative breast cancer cells upregulate NLRP3, whose activation induces the EMT process [218]. CY-09, an antagonist of NLRP3, curtails IL-1β production, EMT, and cell viability [218]. The signal elicited by IL-1β/IL-1R seems to be hostile. In malignant pleural mesothelioma, platinum plus pemetrexed increases IL-1R expression that is correlated with poor overall survival [174]. Accordingly, a synergistic effect is observed in the combined therapy of cisplatin and IL-1R antagonist (Anakinra) against malignant mesothelioma [215]. However, macrophage-derived IL-1β through α-tubulin acetylation after paclitaxel treatment seems to be beneficial in eliciting antibacterial innate responses [219]. It is still elusive whether this paclitaxel-mediated NLRP3 activation is able to facilitate anti-tumor immunity.

**Fig. 4:**
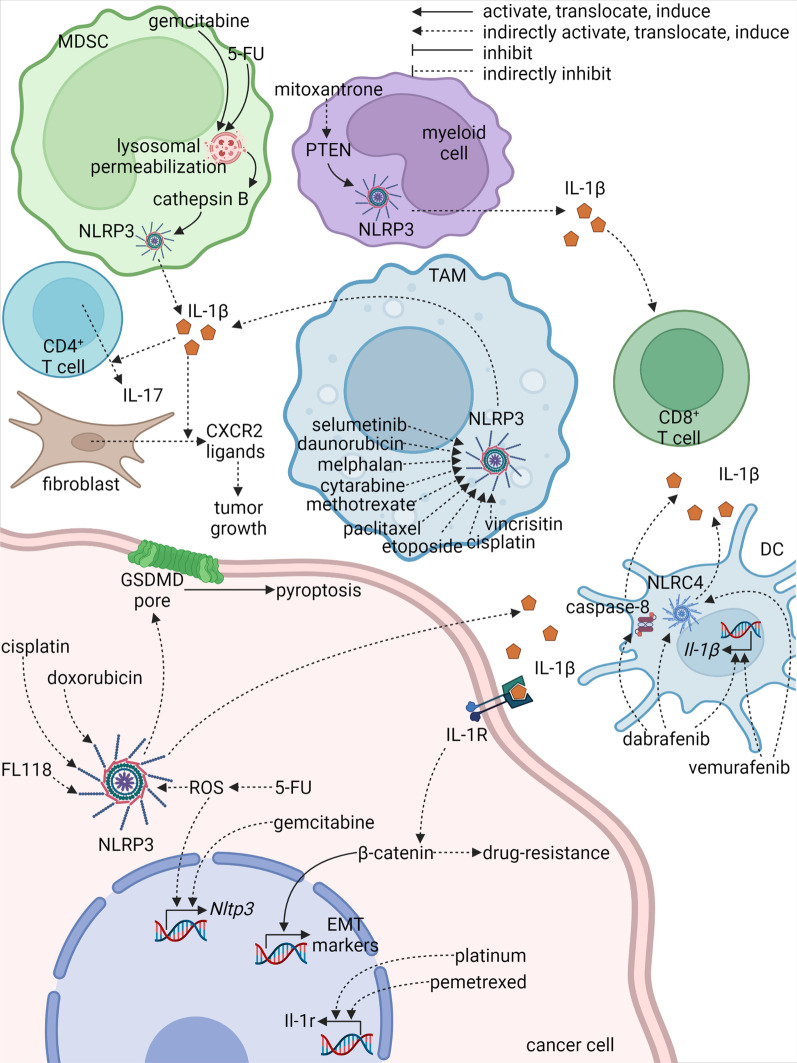
Role of inflammasomes in chemotherapy and target therapy

In summary, inflammasome-induced pyroptosis is involved in the direct cell-killing effects of chemotherapy agents, but the IL-1β/IL-1R signal elicited by these agents seems to be detrimental in most cases. A combination of chemotherapy agents and inhibitors targeting the IL-1β/IL-1R signal might improve the outcome of chemotherapy. The inflammasome-related mechanisms elicited by chemotherapy and target therapy agents in different cell subsets are summarized in Fig. 4.

### Inflammasomes in radiotherapy

Radiotherapy has been applied in various kinds of tumors, whose anti-tumor effects are based on direct DNA damage through radiation and indirect DNA damage through ROS resulting in apoptosis [220]. However, novel findings demonstrate that pyroptosis is also a downstream event of irradiation. After irradiation, activated inflammasomes cause not only pyroptosis but also secretion of IL-1β and IL-18. Most present research works focus on the inflammasome-induced tissue damage, while several others reveal the potential activation of anti-tumor immunity through radiation-induced inflammasomes.

Although many precious technics have been invented to improve radiotherapy, side effects of radiotherapy seem to be ineluctable. Anti-tumor effects of radiation are accompanied by damage of normal tissues, including oral mucositis, skin reaction, lung damage, intestinal injury, hematopoietic failure, and others [102, 221]. Radiation promotes expression of inflammasome components, such as AIM2, NLRP3, caspase-1, caspase-4, IL-1β, and IL-1α [222, 223] facilitating inflammasome activation. AIM2, a sensor of double-stranded DNA fragments, is able to enter the nucleus to detect damaged DNA and initiate inflammasome assembly after irradiation [102]. The activation of AIM2 and the following caspase-1-dependent cell death can be impeded by AIM2 knockout [102] and andrographolide that prevents AIM2 from entering the nucleus [224]. Besides AIM2, NLRP3 is elicited by mitochondrial oxidative stress and bioenergetics impairment [225]. Tissue damages caused by NLRP3 activation [225, 226] can be eliminated via NLRP3 knockout [227], melatonin that protects mitochondria [225], and resveratrol that represses NLRP3 expression through activating Sirtuin 1 [228]. Additionally, caspase-11, a non-canonical inflammasome signal, is also provoked by radiation through cyclic GMP–AMP synthase (cGAS) indicating cross-talk between cGAS and inflammasomes [229]. Downstream mechanisms of AIM2, NLRP3, and caspase-11 include GSDMD-dependent pyroptosis [226, 227] and IL-1β-dependent inflammation [222]. IL-1β is the causative factor for the elevation of neutrophils, lymphocytes, eosinophils, and macrophages [230]. The infiltrated inflammatory cells engender tissue damage such as lung tissue collapse [222] and progressive lung fibrosis [224]. In a word, AIM2, NLRP3, and caspase-11 inflammasomes participate in radiation-induced tissue damage through different mechanisms.

Some scientists have proposed that inflammasomes might get involved in anti-tumor immunity by releasing tumor antigens and activating immune cells [231]. This concept is inspired by the phenomenon that local irradiation harnesses anti-tumor immunity to attack remaining tumor cells [232]. Mechanically, radiotherapy induces cell death, which releases various DAMPs and tumor antigens [233]. DAMPs activate inflammasomes that are able to coordinate with TLR4 signal to induce IL-1β secretion and adaptive anti-tumor immunity in DCs [206]. Thus activating inflammasomes in the context of tumor-derived antigens’ existence may facilitate adaptive anti-tumor immunity after irradiation.

**Fig. 5:**
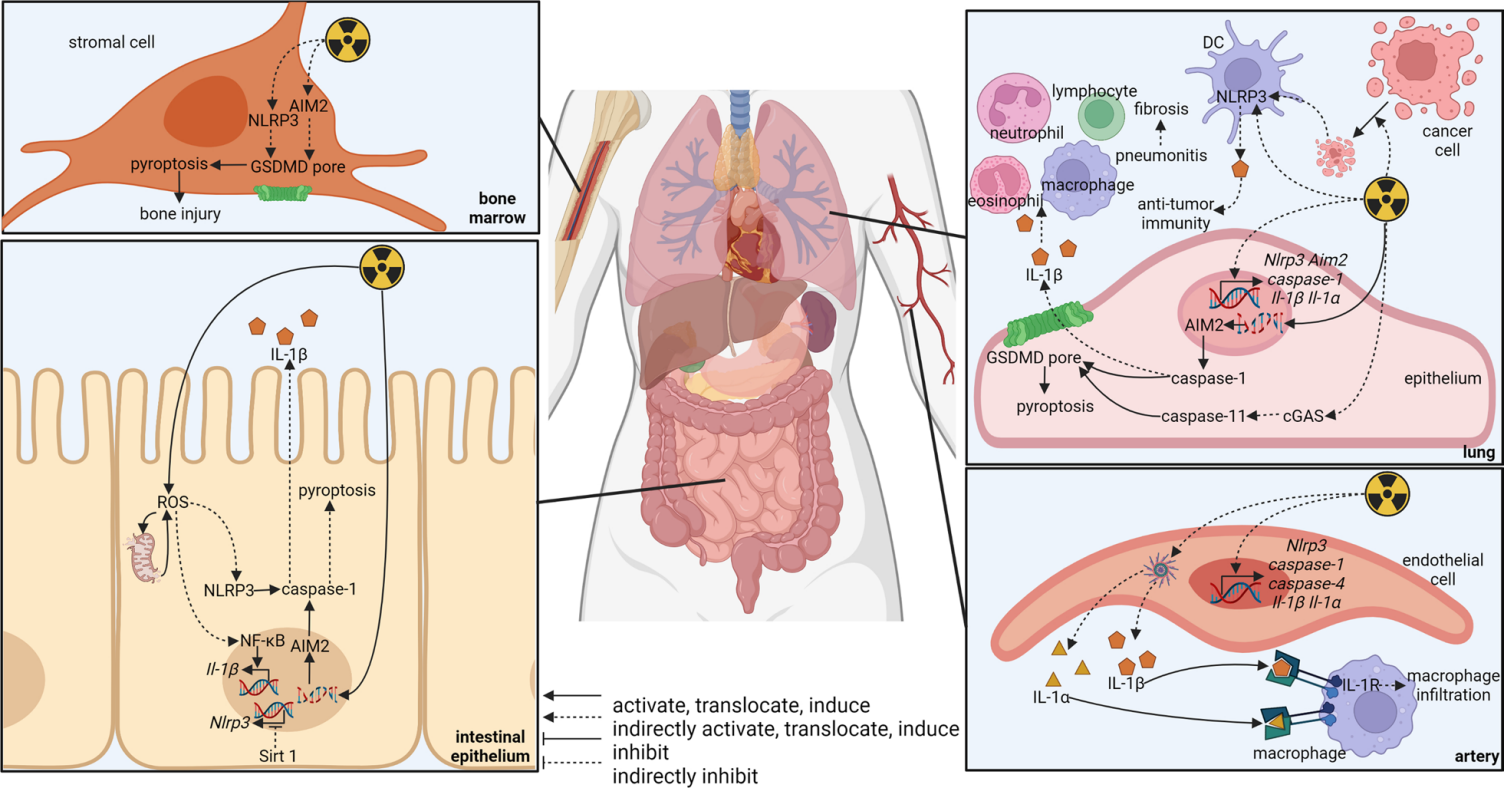
Role of inflammasomes in radiotherapy

Interestingly, inflammasome activation in tumor cells is probably related to radiotherapy resistance. Radiotherapy-resistant breast cancer cell line, MDA-MB-231, shows a higher level of inflammasome activation through TNF-α/ATP/P2Y_2_R pathway than ordinary MDA-MB-231 [175]. Although mRNA of NLRP3, NLRC4, ASC, and caspase-1 are upregulated in radiotherapy-resistant MDA-MB-231, NLRC4/ASC/caspase-1 has been verified to be the main inflammasome activated by TNF-α/ATP/P2Y_2_R pathway [234]. As a result, IL-1β from these radiotherapy-resistant tumor cells accentuates invasion, angiogenesis, and tumor growth [175, 234]. Further research works are needed to elucidate the relationship between inflammasomes and canonical mechanisms of radiotherapy resistance. The roles of inflammasomes in different tumors during radiotherapy are summarized in Fig. 5.

## Inflammasomes in target therapy

In addition to chemotherapy and radiotherapy, targeted therapy agents also have been reported to stimulate inflammasome signals. In melanoma, mitogen activated kinase-like protein (MAPK) inhibitor, selumetinib, initiate IL-1β production from macrophages, which promotes secretion of C-X-C Motif Chemokine Receptor 2 (CXCR2) ligands from fibroblasts leading to enhanced tumor growth and dampened therapeutic effect of MAPK inhibitors [235]. Additionally, NLRC4 in DCs is activated by dabrafenib and vemurafenib, two B-Raf proto-oncogene (BRAF)^V600E^ inhibitors [236]. A high dosage of dabrafenib also activates inflammasomes in a caspase-8-dependent manner [236]. The related mechanisms are summarized in Fig. 4. Limited research works about the activation of inflammasomes by targeted therapy agents are available at present. Instead, more attention has been paid to targeting the inflammasome pathway in tumors.

## Therapies targeting inflammasomes

Knowing the pivotal role of the inflammasome pathway in the progression and therapy of tumors, scientists have developed a host of agents to inhibit or activate inflammasomes. Since the inflammasome pathway is composed of several proteins, there has been plenty of small molecular chemicals and proteins targeting this pathway. These agents can be generally categorized according to their targets [3]. The agents targeting inflammasomes in preclinical and clinical stages are listed in Tables 2 and 3, respectively.

**Table 2:** Preclinical research works about inflammasome interventions

Type of interventions	Agents	Type of diseases	Outcomes	References
NLRP3 inhibitor	MCC950	Colorectal carcinoma	MCC950 reverses FL118-induced pyroptosis	[216]
		Pancreatic cancer	MCC950 abrogates NLRP3/caspase-1/IL-1β-mediated cell proliferation	[159]
		Head and neck squamous cell carcinoma	MCC950 reduces MDSCs, Tregs, and TAMs while increasing CD4^+^ and CD8^+^ T cells in TME where NLRP3 is overexpressed	[203]
	BAY 11–7082	T cell leukemia	Apoptosis is induced in T cell leukemia cells by BAY 11–7082 through inhibiting NF-κB	[297]
		Gastric cancer	Apoptosis is induced in gastric cancer cells by BAY 11–7082 through inhibiting NF-κB	[298]
		Lupus nephritis	Lupus nephritis is attenuated by BAY 11–7082 through inhibiting both NLRP3 and NF-κB	[299]
		Psoriasis	BAY 11–7082 protects animal models from psoriasis through inhibiting both NLRP3 and NF-κB	[300]
	ACT001	Parkinson’s disease	ACT001 ameliorates NLRP3-mediated neuroinflammation in animal models of Parkinson’s disease	[260]
	Isoliquiritigenin	Diet-induced insulin resistance	Isoliquiritigenin inhibits diet-induced insulin resistance through inhibiting NLRP3 activation	[256]
	Tranilast	Gouty arthritis, cryopyrin-associated autoinflammatory syndromes, and type 2 diabetes	Tranilast shows preventive or therapeutic efficacy in three mouse models of NLRP3-related diseases	[250]
		Atherosclerosis	Tranilast dampens the initiation and progression of atherosclerosis through enhancing NLRP3 ubiquitination	[301]
		NSCLC	Tranilast inhibits EMT invasion, and metastasis of lung cancer cell lines	[252]
		Gastric cancer	Tranilast blocks interaction between mesothelial cells and cancer cells resulting in diminished tumor growth and fibrosis	[253]
	OLT1177	Melanoma	OLT1177 disrupts IL-1β/IL-6/STAT3 axis in tumor cells and reduces immunosuppressive activities in MDSCs	[202]
		Melanoma	OLT1177 reduces MDSCs expansion and tumor growth, whose effects could be further improved in combination with anti-PD-1	[245]
		Alzheimer’s disease	OLT1177 reduces the number of plaques in cortex and rescues cognitive impairment	[302]
		Allergic asthma	Both i.p. and oral treatment of OLT1177 alleviate allergic asthma	[303]
	CY-09	Breast cancer	CY-09 curbs NLRP3-mediated drug resistance and EMT	[218]
		Diet-induced hepatic steatosis	CY-09 ameliorates high-fat diet-induced hepatic steatosis	[304]
		Osteoarthritis	CY-09 attenuates osteoarthritis development through inhibiting NLRP3-mediated pyroptosis of chondrocytes	[305]
	MNS	Pancreatic cancer	MNS inhibits cell invasion, migration, and proliferation. Combination of MNS with cytokine-induced killer cells decreases tumor growth	[306]
		Breast cancer	MNS suppresses metastasis properties of cells	[307]
		Burn wound	MNS ameliorates burn wound progression, neutrophil infiltration, and cytokine production by inhibiting NLRP3	[308]
	Oridonin	Peritonitis, gouty arthritis, and type 2 diabetes	Oridonin shows preventive and therapeutic efficacy in three mouse models of NLRP3-related diseases	[255]
		Small cell lung cancer	Oridonin attenuates migration and EMT of cancer cells	[309]
		Oral cancer	Oridonin impedes cell growth	[310]
	Glyburide	Lung cancer	Glyburide attenuates inflammation-related lung tumorigenesis by inhibiting NLRP3	[311]
	BOT-4-one	Urate-induced peritonitis	BOT-4-one shows strong protective effect against urate-induced peritonitis through inhibiting NLRP3	[312]
		Lymphoma	BOT-4-one suppresses proliferation and survival of lymphoma cells	[313]
	Parthenolide	In vitro research	Parthenolide inhibits the activation of NLRP3	[256]
	Glycyrrhizin	In vitro research	Glycyrrhizin inhibits the activation of TLR4, NF-κB, and NLRP3	[256]
	NU9056	In vitro research	NU9056 inhibits NLRP3 activation indirectly through inhibiting KAT5	[79]
	Methylene blue	Spinal cord injury	Methylene blue alleviates neuroinflammation through inhibiting NLRP3	[314]
		In vitro research	Methylene blue is a broad-spectrum inflammasome inhibitor against NLRP3, NLRC4, AIM2, and non-canonical inflammasomes	[261]
	Pioglitazone	Traumatic brain injury	Pioglitazone treatment decreases expression of IL-1β, caspase-1, and NLRP3	[315]
	Fenamate NSAIDs	Alzheimer’s disease	Fenamate NSAIDs show therapeutic effects in Alzheimer’s disease through inhibiting NLRP3	[316]
	Resveratrol	Renal cancer	Resveratrol suppresses tumor progression through downregulating expression of NLRP3	[258]
		Doxorubicin-induced cardiotoxicity	Resveratrol reduces doxorubicin-induced cardiac injury and systemic inflammation	[259]
		Radiation-induced inflammatory bowel disease	Resveratrol alleviates bowel inflammation after irradiation by repressing NLRP3 expression	[228]
	JC-171	Multiple sclerosis	JC-171 delays progression of multiple sclerosis by interfering with NLRP3/ASC interaction	[317]
	JC-124	Alzheimer’s disease	JC-124 inhibits NLRP3 and shows neuroprotective effect	[318]
	Colchicine	Ischemia–reperfusion injury	Colchicine reduces liver damage in mouse model of renal ischemia–reperfusion injury by downregulating NLRP3, caspase-1, and IL-1β	[319]
	IFN39	In vitro research	IFN39 inhibits NLRP3-ASC speckle formation through blocking NEK7-NLRP3 interaction	[320]
	Maxing shigan decoction	*Mycoplasma pneumonia* infection	Maxing shigan decoction suppresses NLRP3-induced cell pyroptosis and IL-1β production after *Mycoplasma pneumonia* infection	[321]
	Sulforaphane	In vitro research	Sulforaphane attenuates activation of both NLRP3 and NLRC4	[262]
		Ischemia–reperfusion injury	Sulforaphane reduces retinal ischemia–reperfusion injury and reduces retinal ganglion cell death	[322]
	β-hydroxybutyrate	Glioma	NLRP3-mediated migration of glioma cells is suppressed by β-hydroxybutyrate	[323]
		Alzheimer’s disease	Pathology of Alzheimer’s disease is alleviated by β-hydroxybutyrate through inhibiting NLRP3	[324]
		Gout flares	Gout flares is relieved by β-hydroxybutyrate through inhibiting NLRP3 in neutrophil	[325]
	16,673-34-0	Cardiac dysfunction	Western diet-induced cardiac dysfunction is prevented by 16,673-34-0	[326]
		Ischemia–reperfusion injury	Heart ischemia–reperfusion injury is reduced by 16,673-34-0	[327]
	Celastrol	Melanoma	Celastrol inhibits migration and invasion of melanoma cells by suppressing macrophage-derived NLRP3/IL-1β pathway	[172]
	NBC series	In vitro research	NBC series inhibits activation of NLRP3 without affecting Ca^2+^ homeostasis	[328]
	Apigenin	Peritonitis	Apigenin ameliorates inflammatory symptoms related to NLRP3 activation	[329]
	Fc11a-2	Colitis	Fc11a-2 attenuates symptoms and secretion of pro-inflammatory cytokines in colitis by targeting NLRP3	[330]
	Formononetin	Colitis	Formononetin prevents colonic cell injury by reducing NLRP3, ASC, and IL-1β protein levels	[331]
	Triptolide	Myocardial remodeling	Triptolide attenuates myocardial remodeling by targeting NLRP3	[332]
	Andrographolide	Colitis-associated cancer	Andrographolide reduces colitis and tumor burden by inhibiting NLRP3 and triggering mitophagy	[333]
	Curcumin	Colitis	Curcumin ameliorates colitis symptoms by inhibiting NLRP3 in macrophages	[334]
	Quinazolin-4(3H)-ones	In vitro research	Quinazolin-4(3H)-ones inhibits NLRP3-mediated IL-1β release in monocyte	[335]
	Arglabin	Atherosclerosis	Arglabin shows anti-atherogenic effects partially through targeting NLRP3	[336]
	LFG-500	NSCLS	LFG-500 suppresses NLRP3-mediated EMT, migration, and metastasis	[178]
	ibrutinib	In vitro research	Ibrutinib indirectly inhibits NLRP3 inflammasomes by suppressing phosphorylated BTK that directly inhibited NLRP3 and ASC	[257]
AIM2 inhibitor	Andrographolide	Radiation-induced lung inflammation	Andrographolide prevents AIM2 from entering the nucleus	[224]
	glycyrrhizin	In vitro research	Glycyrrhizin inhibits activation of AIM2	[256]
	Methylene blue	In vitro research	Methylene blue is a broad-spectrum inflammasome inhibitor against NLRP3, NLRC4, AIM2, and non-canonical inflammasomes	[261]
NLRC4 inhibitor	Sulforaphane	In vitro research	Sulforaphane attenuates activation of both NLRP3 and NLRC4	[262]
	Methylene blue	In vitro research	Methylene blue is a broad-spectrum inflammasome inhibitor against NLRP3, NLRC4, AIM2, and non-canonical inflammasomes	[261]
ASC inhibitor	CRID3 (MCC950)	In vitro research	CRID3 directly interacts with ASC causing blocked formation of ASC specks	[271]
		Spinal cord injury	CRID3 improves histology and behavior results after spinal cord injury by inhibiting ASC-related inflammasomes	[270]
	ibrutinib	In vitro research	Ibrutinib indirectly inhibits NLRP3 inflammasomes by suppressing phosphorylated BTK that directly inhibits NLRP3 and ASC	[257]
Caspase-1 inhibitor	VX-765	NSCLC	ROS/NF-κB/NLRP3/GSDMD axis-induced pyroptosis is inhibited by VX-765	[263]
		NSCLC	VX-765 inhibits cell migration by blocking AIM2 signal	[264]
		Alzheimer’s disease	VX-765 alleviates neuropathology and cognitive impairment in mouse model of Alzheimer’s disease	[337]
	VX-740	Colitis	VX-740 mitigates IL-1β secretion in dextran sodium sulfate-induced colitis	[338]
		Osteoarthritis	VX-740 attenuates joint damage in mouse models of osteoarthritis	[339]
	VRT-018858	Transient ischemia	VRT-018858 shows protective effect against brain damage in transient ischemia	[340]
	Thalidomide	Melanoma	Thalidomide reduces tumor growth through inhibiting caspase-1 in MDSCs	[267]
	Ac-YVAD-CHO	Melanoma	Ac-YVAD-CHO inhibits iNOS-induced apoptosis	[268]
		Melanoma	Ac-YVAD-CHO inhibits phloretin-induced apoptosis	[269]
	Ac-FLTD-CMK	Traumatic brain injury	Ac-FLTD-CMK shows neuroprotective effect in traumatic brain injury through inhibiting pyroptosis	[341]
	Ac-YVAD-CMK	Hepatocellular carcinoma	Ac-YVAD-CMK reverses caspase-1-mediated pyroptosis	[265]
		Breast cancer	Ac-YVAD-CMK increases proliferation and invasion, while decreasing apoptosis in cancer cells	[342]
		Renal cancer	Ac-YVAD-CMK abrogates AIM2-mediated anti-tumor effect	[343]
	Z-YVAD-fmk	Prostate cancer	Z-YVAD-fmk inhibits radiation-induced apoptosis by targeting caspase-1	[266]
	Q-VD-OPh	Leukemia	Q-VD-OPh in combination with vitamin D show anti-leukemia effects through inducing differentiation	[344]
IL-1α inhibitor	Lutikizumab	In vitro research	Lutikizumab specifically binds to IL-1α and IL-1β simultaneously	[345]
IL-1β inhibitor	Canakinumab	rheumatoid arthritis	Canakinumab is effective in joint inflammation models	[278]
	Rilonacept	autoimmune disorders	High-affinity “cytokine traps” potently block cytokines in vitro and in vivo	[279, 280]
	Gevokizumab	Heart failure	Gevokizumab limits cardiac remodeling and coronary dysfunction	[281]
	Lutikizumab	In vitro research	Lutikizumab specifically binds to IL-1α and IL-1β simultaneously	[345]
IL-18 inhibitor	IL-18BP	Colorectal carcinoma	IL-18BP binds to IL-18 with high-affinity limiting anti-tumor immunity	[282]
IL-1R inhibitor	Anakinra	Burkitt lymphoma	Anakinra abrogates cytokine release syndrome during CAR-T therapy	[289]
		Breast cancer	Anakinra reduces tumor growth by abrogating IL-22 production	[283]
		Mesothelioma	Synergistic effect is observed in combined of cisplatin and Anakinra against malignant mesothelioma	[215]
	AMG 108	Osteoarthritis	AMG 108 decreases neutrophil count	[346]
GSDMD inhibitor	Necrosulfonamide	NSCLC	ROS/NF-κB/NLRP3/GSDMD axis-induced pyroptosis is inhibited by necrosulfonamide	[263]
		Alzheimer’s disease	Necrosulfonamide inhibits β-amyloid-induced neuronal pyroptosis	[347]
	LDC7559	In vitro research	LDC7559 binds to GSDMD and inhibits NETosis	[272]
	Disulfiram	Breast cancer	Metabolite of disulfiram shows anticancer effect	[348]
		Nasopharyngeal cancer	Disulfiram/copper shows potent cytotoxic effects on cancer cells and fibroblasts	[275]
		Sepsis	Disulfiram prevents NET release from neutrophils leading to reduced multiple organ dysfunction	[273]
		In vitro research	Disulfiram inhibits GSDMD pore formation	[274]
	Bay 11–7082	Multiple myeloma	Bay 11–7082 induces apoptosis in multiple myeloma cells	[276, 277]
		In vitro research	Bay 11–7082 inhibits GSDMD pore formation	[274]
NLRP3 activator	Polyphyllin VI	NSCLC	Polyphyllin VI induces pyroptosis through activating NLRP3	[263]
	17β-estradiol	Hepatocellular carcinoma	17β-estradiol provokes pyroptosis via NLRP3	[265, 290]
	BMS-986299	Cancer	BMS-986299 shows potential anticancer effects	[3]

**Table 3:** Inflammasome interventions in tumor-related clinical trials

Interventions related to inflammasomes	Diseases	Stage of development	Comments	NCT numbers
Glycyrrhizin	Lung cancer	I/II (completed)	NLRP3 inhibitor	NCT02449122
	Liver cancer	I/II (completed)	NLRP3 inhibitor	NCT02449109
ACT001	Glioblastoma	I/II (recruiting)	NLRP3 inhibitor	NCT05053880
Andrographolides	Colon cancer	II (terminated)	NLRP3/AIM2 inhibitor	NCT01993472
Methylene Blue	Colon cancer	III (completed)	broad-spectrum inflammasome inhibitor	NCT01694966
Methylene blue	Breast cancer	Not Applicable	broad-spectrum inflammasome inhibitor	NCT02084784
BMS-986299	Advanced cancer	I (terminated)	NLRP3 activator	NCT03444753
Thalidomide	Prostate cancer	II (completed)	caspase-1 inhibitor	NCT00400517
Thalidomide	Multiple myeloma	III (completed)	caspase-1 inhibitor	NCT01296503
Canakinumab	Lung cancer	III (completed)	IL-1β inhibitor	NCT01327846
Canakinumab	Lung cancer	III (active, not recruiting)	IL-1β inhibitor	NCT 03,447,769
Canakinumab	Lung cancer	II (recruiting)	IL-1β inhibitor	NCT04905316
Canakinumab	Lung cancer	III (active, not recruiting)	IL-1β inhibitor	NCT03631199
Canakinumab	Lung cancer	II (completed)	IL-1β inhibitor	NCT 03,968,419
Canakinumab	Lung cancer	II (recruiting)	IL-1β inhibitor	NCT04789681
Canakinumab	Lung cancer	III (completed)	IL-1β inhibitor	NCT03626545
Canakinumab	Lung cancer	I (terminated)	IL-1β inhibitor	NCT03064854
Canakinumab	Lung cancer, breast cancer, colon cancer	I (completed)	IL-1β inhibitor	NCT02900664
Canakinumab	Pancreatic cancer	I (active, not recruiting)	IL-1β inhibitor	NCT04581343
Canakinumab	Pancreatic cancer	III (recruiting)	IL-1β inhibitor	NCT04229004
Canakinumab	Breast cancer	I (active, not recruiting)	IL-1β inhibitor	NCT03742349
Canakinumab	Myelodysplastic syndromes and chronic myelomonocytic leukemia	II (recruiting)	IL-1β inhibitor	NCT04239157
Canakinumab	Myelodysplastic syndromes	II (recruiting)	IL-1β inhibitor	NCT05237713
Canakinumab	Myelodysplastic syndromes	I/II (recruiting)	IL-1β inhibitor	NCT04798339
Canakinumab	Myelodysplastic syndromes	I (recruiting)	IL-1β inhibitor	NCT04810611
Canakinumab	Renal cancer	Early Phase 1 (recruiting)	IL-1β inhibitor	NCT04028245
Canakinumab	Melanoma	II (active, not recruiting)	IL-1β inhibitor	NCT03484923
Canakinumab	Clonal cytopenias of unknown significance	II (recruiting)	IL-1β inhibitor	NCT05641831
Gevokizumab	Colon cancer	I (active, not recruiting)	IL-1β inhibitor	NCT03798626
Tadekinig alfa (IL-18BP)	CAR T-cell-related cytokine release syndrome	Early Phase 1 (recruiting)	IL-18 inhibitor	NCT05306080
Xilonix	Colon cancer	III (terminated)	IL-1α inhibitor	NCT01767857
Xilonix	Colon cancer	III (completed)	IL-1α inhibitor	NCT02138422
Xilonix	Pancreatic cancer	I (completed)	IL-1α inhibitor	NCT03207724
Xilonix	Advanced cancer	I (completed)	IL-1α inhibitor	NCT01021072
SB-485232	Ovarian cancer	I (completed)	Recombinant IL-18	NCT00659178
SB-485232	Melanoma	II (completed)	Recombinant IL-18	NCT00107718
SB-485232	Lymphoma	I (completed)	Recombinant IL-18	NCT00500058
SB-485232	Lymphoma	I (completed)	Recombinant IL-18	NCT01768338
SB-485232	Solid tumor	I (completed)	Recombinant IL-18	NCT00085878
SB-485232	Solid tumor and lymphoma	I (completed)	Recombinant IL-18	NCT00085904
ST-067	Solid tumor	I/II (recruiting)	Recombinant IL-18	NCT04787042
huCART19-IL18	Leukemia and lymphoma	I (recruiting)	CAR-T targeting CD19 and expressing IL-18	NCT04684563
Anakinra	Breast cancer	I (completed)	IL-1 receptor inhibitor	NCT01802970
Anakinra	Rectal cancer	I (recruiting)	IL-1 receptor inhibitor	NCT04942626
Anakinra	Colon cancer	II (completed)	IL-1 receptor inhibitor	NCT02090101
Anakinra	Pancreatic cancer	I (unknown)	IL-1 receptor inhibitor	NCT02021422
Anakinra	Pancreatic cancer	Early I (completed)	IL-1 receptor inhibitor	NCT02550327
Anakinra	Pancreatic cancer	II (not yet recruiting)	IL-1 receptor inhibitor	NCT04926467
Anakinra	Prostate cancer	I (active, not recruiting)	IL-1 receptor inhibitor	NCT04227275
Anakinra	Multiple myeloma and plasma cell neoplasm	II (completed)	IL-1 receptor inhibitor	NCT00635154
Anakinra	Multiple myeloma	I/II (active, not recruiting)	IL-1 receptor inhibitor	NCT03430011
Anakinra	Multiple myeloma	II (completed)	IL-1 receptor inhibitor	NCT03233776
Anakinra	Multiple myeloma	II (recruiting)	IL-1 receptor inhibitor	NCT04099901
Anakinra	Myeloma	I (completed)	IL-1 receptor inhibitor	NCT02492750
Anakinra	B-cell lymphoma	II (active, not recruiting)	IL-1 receptor inhibitor	NCT04432506
Anakinra	B-cell lymphoma	II (recruiting)	IL-1 receptor inhibitor	NCT04359784
Anakinra	B-cell lymphoma	II (recruiting)	IL-1 receptor inhibitor	NCT04205838
Anakinra	Lymphoma	II (active, not recruiting)	IL-1 receptor inhibitor	NCT04150913
Anakinra	B-cell lymphoma/leukemia	II (recruiting)	IL-1 receptor inhibitor	NCT04148430
Anakinra	Chronic lymphocytic leukemia	I (unknown)	IL-1 receptor inhibitor	NCT04691765
Anakinra	Solid tumor	I (completed)	IL-1 receptor inhibitor	NCT00072111
Anakinra	Advanced Cancer	I (completed)	IL-1 receptor inhibitor	NCT01624766

Although agents targeting different inflammasome components are available, it must be carefully determined whether upstream or downstream proteins of inflammasomes should be chosen as the target of interventions. Agents targeting IL-1α, IL-1β, IL-18, and IL-1Rs have shown promising therapeutic effects in various inflammasome-related diseases during clinical trials [237–239]. Systematic administration of IL-1 signal inhibitors is a direct strategy, but long-term blockage of IL-1 signal increases the risk for serious infection [240, 241], because this strategy eliminates pro-inflammatory IL-1 signal roughly, which is crucial for innate and adaptive immunity. In contrast, targeting upstream proteins might improve the precision of the intervention of inflammasomes. For example, vigorous activation of NLRP3 has been demonstrated in atherosclerosis [237, 242], thus deterring NLRP3 is a feasible intervention [243] without interfering with other inflammasome sensors AIM2, NLRP1, NLRC4, and Pyrin that are responsible for detecting bacteria and virus infection. However, this strategy might be less effective than targeting the IL-1 signal owing to the potential role of AIM2 in atherosclerosis development [244]. Thus, the advantages and disadvantages of targeting upstream or downstream molecules should be extensively evaluated in order to optimize our choices.

As for inflammasome interventions in tumors, specific delivery of agents may be crucial, because inflammasomes in different cell subsets show diverse effects sometimes [117, 196, 202, 206]. AIM2 from TAMs [196] and NLRP3 from DCs [206] elicit anti-tumor immune response indicating the therapeutic potential of specifically delivering correspondence activators. Tumor cell-derived inflammasome signals facilitate tumor development [202, 245] indicating inflammasome inhibitors targeting tumor cells to be beneficial. At present, few attempts have been made to specifically activate inflammasome in certain cell subset in TME. This attempt may help understand the conflicting effects of inflammasomes from different cell subsets on tumor behaviors and improve the therapeutic effects of inflammasome interventions.

### NLRP3 inhibitors

Given the diverse roles of NLRP3 in various diseases, it is not surprising that most attention has been focused on developing NLRP3 inhibitors. In many cases, NLRP3 inhibitors are initially invented for the treatment of non-malignant diseases, then these agents are found to be effective in tumor therapies [159, 203, 246]. These agents inhibit NLRP3 in different mechanisms, some of which remain elusive.

The most commonly used NLRP3 inhibitor in preclinical experiments is MCC950 (CRID3), which inhibits NLRP3 with nM potency without interfering with other inflammasome sensors [246]. Mechanistically, MCC950 interacts with Walker B motif of NACHT domain that is close to the ATP binding pocket, thereby blocking the hydrolysis of ATP and suppressing NLRP3 activation [247]. This specific blockage is consistent no matter whether in wild-type or mutated NLRP3 [248]. Another structural research has illustrated that the sulfonylurea group of MCC950 interacts with the Walker A motif of NLRP3 and it is sandwiched between Arg351 and Arg578 resulting in stabilized NACHT and LLR domains relative to each other [63]. MCC950 is initially developed as a potential therapeutic agent for CAPS, as well as other autoinflammatory and autoimmune diseases [246]. Later research works illustrate the potential anti-tumor effect of MCC950 against pancreatic cancer and head and neck squamous cell carcinoma [159, 203]. Similar to MCC950, the target of CY-09 is Walker A motif of NACHT, which binds ATP [249]. Another inhibitor that targets NACHT domain is tranilast [250]. However, it also suppresses TGF-β, MAPK, and NF-κB signals [251]. Present results have demonstrated that tranilast inhibits malignant behaviors of NSCLC and gastric cancer [252, 253], but the authors do not clarify whether these effects are related to inhibited inflammasome signal. Other inhibitors targeting NACHT domain include 3,4-methylenedioxy-β-nitrostyrene (MNS) [254] and oridonin [255]. MNS also binds to LRR domain [254]. Interestingly, isoliquiritigenin and glycyrrhizin are able to inhibit NLRP3 through both signal 1 (TLR4) and signal 2 (NLRP3) [256], but their inhibitory potency is not as powerful as that of MCC950. There is also an indirect NLRP3 inhibitor, ibrutinib [257]. Ibrutinib inhibits the generation of phosphorylated Bruton tyrosine kinase (BTK) that directly interacts with NLRP3 and ASC leading to the formation of inflammasomes [257]. Another indirect NLRP3 modulator is resveratrol that suppresses the expression of NLRP3 in renal cancer cells [258]. In a word, present findings imply that NACHT domain is the key target for inhibitors.

Although NLRP3 inhibitors alone have shown anti-tumor effects, some attempts highlighted the potential combination of NLRP3 inhibitors with other therapeutic methods. OLT1177 disrupts IL-1β/IL-6/STAT3 axis in TME resulting in reduced tumor growth through attenuating immunosuppressive activities in MDSCs [202], and the anti-tumor effect is further enhanced in combination with anti-PD-1 [245]. In addition to enhancing therapeutic effects, NLRP3 inhibitors may protect against the side effects of chemotherapy and radiotherapy. Resveratrol reduces doxorubicin-induced cardiac injury and systemic inflammation through downregulating NLRP3 inflammasomes [259]. Likewise, bowel inflammation after irradiation is also suppressed by resveratrol through a similar mechanism [228]. More research works are needed to explore other possibilities of such a combination.

It is suppressing that although many kinds of NLRP3 inhibitors have been invented, only a few of these agents have entered clinical trials for tumor therapy. ACT001 combined with anti-PD-1 or ACT001 alone has been applied for a phase I/II trial against glioblastoma. This agent is primarily developed for Parkinson’s disease [260]. Others are some agents that have been reported to inhibit NLRP3, including glycyrrhizin and andrographolides. Whether their anti-tumor effects are underpinned by NLRP3 inhibition remains to be further evaluated.

### AIM2 inhibitors

Compared with NLRP3, limited AIM2 inhibitors have been found. Two agents are able to inhibit AIM2, but they are not specific inhibitors. Glycyrrhizin suppresses both AIM2 and NLRP3 [256]. Methylene blue is a broad-spectrum inflammasome inhibitor against NLRP3, NLRC4, AIM2, and non-canonical inflammasomes [261]. Fortunately, andrographolide has shown a promising effect for the future clinical application that it reduces radiation-induced lung inflammation and fibrosis by preventing AIM2 from entering the nucleus and sensing DNA damage [224]. Although potential anti-tumor effects of glycyrrhizin, andrographolide, and methylene blue would be evaluated in colon cancer, breast cancer, and liver cancer during clinical trials, specific AIM2 inhibitors are in need.

### NLRC4 inhibitors

Few specific NLRC4 inhibitors are available. Instead, two inflammasome inhibitors with limited selectivity have been reported to suppress NLRC4. Sulforaphane attenuates the activation of both NLRC4 and NLRP3 at μM potency, which limits inflammation during peritonitis [262]. Methylene blue, a broad-spectrum inflammasome inhibitor, blocks NLRC4, NLRP3, AIM2, and non-canonical inflammasomes, which improves the survival rate of mice challenged with LPS [261]. Considering that many NLRP3 inhibitors target NACHT and that NACHT domain also exists in the NLRC4 inflammasomes, future selective NLRC4 inhibitors might be the derivate of the NLRP3 inhibitors.

### Caspase-1 inhibitors

Owing to the upsurge of the study in caspase-related signals, many inhibitors targeting caspase family members have been developed, some of which are able to inhibit caspase-1. As the inhibitors of the common downstream protein of inflammasomes, caspase-1 inhibitors restrain not only NLRP3-derived but also AIM2-derived inflammasome signals. For example, VX-765 inhibits NLRP3/caspase-1/GSDMD-induced pyroptosis in NSCLC [263], and it also attenuates AIM2-mediated cell migration in NSCLC [264]. An interesting question is whether caspase-1 inhibitors promote or suppress cancer cell growth. Direct inhibition of pyroptosis has been reported in NSCLC by VX-765 [263], liver cancer by Ac-YVAD-CMK [265], and prostate cancer by Z-YVAD-fmk [266]. On the contrary, the caspase-1 inhibitor, thalidomide, impedes tumor growth in melanoma by suppressing caspase-1 in MDSCs [267]. Thus, non-selective administration of caspase-1 inhibitors may promote tumor growth, while selective caspase-1 inhibition in MDSCs may attenuate tumor development. Of note, in the early years when inflammasome signal was not intensively studied, caspase-1 mediated cell death was regarded as apoptosis [268, 269]. These findings should be updated to clarify the kind of cell death. Additionally, the relationship between pyroptosis of cancer cells and tumor growth should be further studied. Because DAMPs from dead cancer cells may elicit inflammasomes in adjacent myeloid cells and probably cancer cells resulting in the recruitment of MDSCs that facilitate tumor growth. At present, thalidomide alone or plus other agents have entered clinical evaluation against multiple myeloma, prostate cancer, and other advanced cancer.

### ASC inhibitors

Although MCC950 has been demonstrated to selectively block the NACHT domain of NLRP3, it is also able to downregulate protein expression of ASC, caspase-1, IL-1β, and IL-18 [270]. This is an in vivo study that tests protein expression in tissues. Thus, it is possible that MCC950 directly inhibits NLRP3-mediated pyroptosis and IL-1β and IL-18 secretion causing reduced infiltration of macrophages [270]. Decreased number of macrophages in tissues may explain the downregulated protein levels of the inflammasome components. However, another research has found that MCC950 inhibits both NLRP3 and AIM2-derived inflammasome formation [271]. The MCC950-mediated ASC suppression is possibly through Glutathione *S*-Transferase Omega 1 (GSTO1), a putative target of MCC950 [271]. In a word, there is a lack of selective and direct ASC inhibitors.

### GSDMD inhibitors

GSDMD pores are the direct cause of pyroptosis and the exit for intracellular mature IL-1β and IL-18. In addition to blocking pyroptosis and secretion of pro-inflammatory cytokines, two GSDMD inhibitors, LDC7559 [272] and Disulfiram [273], also restrain inflammation through curbing NETosis, a special kind of cell death of neutrophils. Although GSDMD inhibitors, disulfiram and Bay 11–7082, potently suppress pyroptosis [274], they show anti-tumor effects through inducing ferroptosis [275] (by disulfiram) or apoptosis [276, 277] (by Bay 11–7082). In a word, the anti-inflammation effects of GSDMD inhibitors have been repeatedly proven, but their applications in tumor development remain to be further evaluated.

### IL-1 signal inhibitors

Four targets of the IL-1 signal have been developed, including IL-1 receptor, IL-1α, IL-1β, and IL-18 that can be intervened by antagonists, antibodies, and binding proteins. These potent anti-inflammatory inhibitors are pleiotropic agents applied in various inflammation-related diseases, for example, rheumatoid arthritis [278] (by canakinumab), autoimmune disorders [279, 280] (by rilonacept), and cardiac remodeling [281] (by gevokizumab). Blocking IL-1 signals might promote or inhibit tumor development. IL-18BP, a binding protein targeting IL-18, limits anti-tumor immunity [282]. However, anakinra, an IL-1 receptor antagonist, reduces IL-1β and downstream production of cancer-promoting IL-22 [283]. Similarly, anti-inflammatory therapy in patients with atherosclerosis by canakinumab reduces lung cancer incidence [284]. This effect has been proven to be underpinned by the reduced tumor-promoting inflammation [285]. For tumor therapies, anakinra gives rise to cytotoxic/NK cell transcriptional pathways and hampers innate inflammation in breast cancer patients receiving chemotherapy [286]. Additionally, anakinra is reported to limit the mucosal barrier injury and the accompanying clinical symptoms induced by melphalan [287]. Although more frequent fatal infections and sepsis are recorded in the canakinumab treatment group, all-cause mortality does not differ significantly between the placebo and the canakinumab group [284]. On the contrary, the anakinra treatment seems to be more safety [286], and no adverse events or dose-limiting toxicities have been observed [287]. In another phase 2 clinical trial, anakinra is applied in patients receiving 5-FU plus bevacizumab therapy, and no grade 4/5 toxicity related to therapy occurs during the study [288]. Interestingly, anakinra abrogates cytokine release syndrome during CAR-T therapy implying its compelling clinical application [289]. A series of clinical trials testing the prevention of CAR-T cell-mediated toxicity by anakinra have been launched, such as NCT04432506, NCT04150913, and NCT04148430. At present, most interventions targeting inflammasome pathways for cancer therapies listed in Table 3 are based on IL-1 signal inhibitors, possibly owing to the ready-made agents for other non-malignant diseases. For example, the therapeutic effects of canakinumab in lung cancer, colon cancer, breast cancer, pancreatic cancer, renal cancer, and leukemia would be evaluated in a number of clinical trials. It is a compelling topic to test whether the combination of these IL-1 signal inhibitors with other therapies can be beneficial for patients.

### Inflammasome activators

Although many results support that activated inflammasomes show anti-tumor effects directly through inducing pyroptosis and indirectly through stimulating immune cells, limited inflammasome activators are developed at present. Polyphyllin VI induces pyroptosis by activating NLRP3 in NSCLC cells [263]. Similarly, 17β-estradiol provokes pyroptosis via NLRP3 in liver cancer cells [265, 290]. Another NLRP3 activator, BMS-986299, shows potential anticancer effects, but the details are largely unknown [3]. BMS-986299 have entered a phase I trial to explore its safety and effectiveness in patients with solid tumor or advanced tumor. An alternative strategy is to supply the downstream IL-1 cytokines directly. Since IL-18 is likely to be beneficial for anti-tumor immunity [182, 282], recombinant IL-18 has been applied in several clinical trials such as NCT00659178, NCT00107718, and NCT00500058. In the clinical trial NCT04684563, CAR-T cells targeting CD19 and expressing IL-18 are applied in patients with chronic lymphocytic leukemia or non-Hodgkin lymphoma. Present results indicate that more efforts should be paid to develop inflammasome activators. Considering that inflammasomes may initiate pyroptosis in tumor cells and that IL-1β and IL-18 have been shown to activate T cells and NK cells, inflammasome activators may improve the effects of immune checkpoint inhibitors.

## Conclusions

In this review, we summarize the mechanisms that activate canonical and non-canonical inflammasome pathways. More importantly, we discuss the roles of canonical and non-canonical inflammasomes in tumorigenesis, tumor cell death, tumor metastasis, immune evasion, chemotherapy, and radiotherapy. Finally, we review the interventions targeting the inflammasome pathways in preclinical and clinical stages.

A good question is how the inflammasomes are activated in TME. Expression levels of inflammasome components have been compared between healthy and tumor tissues [117, 134, 138, 170, 183]. Mice deficient in certain components of the inflammasome pathway [138, 182, 183] or inflammasome inhibitors [196, 291, 292] have been applied to reveal the various influences of inflammasomes on tumor behaviors. Inflammasome activators (such as ATP, H_2_O_2_, monosodium urate, and Mycoplasma hyorhinis) have been used in vitro to confirm that inflammasomes can be activated in certain cell subsets [117, 172, 175, 184]. However, little is known about the direct activators of inflammasomes in TME during tumor progression. The activators may be bacteria [130], cell debris [117], ATP [206], PKR [199], other unknown factors in TME, or more complicated cross-talk between cells. Novel techniques such as single-cell sequencing may improve our understanding of the details during inflammasome activation.

The inflammasome signal seems to be a conserved pathway, which even exists in bacteria [293]. Although similar mechanisms have been identified in different species, discrepancies in *NLR* homologous genes and inflammasome pathways between humans and mice have been found [294]. For example, *Francisella tularensis* activates NLRP3 in humans instead of mice [295]. Thus, more detailed comparisons are needed to answer the question of to what extent can the findings from mouse models be extended to human patients.

It seems fuzzy that inflammasome signals have conflicting effects in different research works. A possible explanation is that inflammasomes can be activated at different extents, which may result in distinct inflammation responses [16, 42]. Future research works should compare the outcomes of different extents of inflammasome activation in various cell subsets in TME. Through this way, we can make accurate decisions about whether and how inflammasomes should be activated or inhibited.

GSDMD-mediated pyroptosis is involved in cancer cell death during chemotherapy and radiotherapy; however, secreted IL-1β may recruit immunosuppressive cell subsets and initiate inflammation-related side effects. Thus, the combination of IL-1R signal inhibitors and chemotherapy or radiotherapy may improve outcomes. On the other hand, NLRP3 in DCs [206] and AIM2 in macrophages [196] have been shown to facilitate anti-tumor immunity. The combination of NLRP3 or AIM2 activators and immune checkpoint inhibitors is a compelling strategy for immunotherapy.

## Data Availability

The materials supporting our conclusion of this review are included within the article.

## Abbreviations

DAMPs: Damage-associated molecular patterns
PAMPs: Pathogen-associated molecular patterns
IL-1: Interleukin-1
GSDMD: Gasdermin D
PRRs: Pattern recognition receptors
TLRs: Toll-like receptors
NLRs: Nucleotide-binding leucine-rich repeat receptors
AIM2: Absent in melanoma 2
NLRP1: NACHT, leucine-rich repeat and pyrin domain-containing 1
NLRP3: NOD-, LRR-, and pyrin domain-containing 3
NAIP: NLR family apoptosis inhibitory protein
NLRC4: NLR family CARD domain-containing 4
ASC: Apoptosis-associated speck-like protein-containing CARD
PYD–PYD: Pyrin–pyrin
ESCRT: Endosomal sorting complex required for transport
LPS: Lipopolysaccharide
dectin-1: Dendritic cell-associated C-type lectin-1
DCs: Dendritic cells
Th: T helper
NK: Natural killer
LRR: Leucine-rich repeat
LeTx: Lethal toxin
DDP: Dipeptidyl peptidase
NF-κB: Nuclear factor-kappa B
TNF-α: Tumor necrosis factor-α
JNK-1: C-Jun N-terminal kinase-1
MyD88: Myeloid differentiation factor 88
mtROS: Mitochondria reactive oxygen species
BRCC3: BRCA1/BRCA2-containing complex 3
KAT5: Lysine acetyltransferase 5
NEK7: NIMA-related kinase 7
CAPS: Cryopyrin-associated periodic syndrome
T3SS: Type III secretion system
T4SS: Type IV secretion system
HIN200: Hematopoietic interferon-inducible nuclear antigens with 200 amino acid repeat
PKN1: Protein kinase N1
PKN2: Protein kinase N2
TME: Tumor microenvironment
SMAD: Small mothers against decapentaplegic
c-myc: V-myc myelocytomatosis viral oncogene homolog
TP53: Tumor protein p53
bcl-2: B-cell lymphoma-2
Bax: Bcl-2-associated X protein
gp130: Glycoprotein 130
STAT3: Signal transducer and activator of transcription 3
MDSCs: Myeloid-derived suppressor cells
IFN-γ: Interferon gamma
4-NQO: 4-Nitroquinoline 1-oxide
NSCLC: Non-small cell lung cancer
EGFR: Epidermal growth factor receptor
ERK: Extracellular signal-regulated kinase
PI3K: Phosphatidylinositol 3-kinase
HIF-1$\mathrm{\alpha }$: Hypoxia-inducible factor-1$\mathrm{\alpha }$
CXCL2: C-X-C motif chemokine ligand 2
TAMs: Tumor-associated macrophages
VEGF: Vascular endothelial growth factor
S1PR1: S1P receptor 1
Vegfa: Vascular endothelial growth factor A
BRCA1: Breast cancer susceptibility gene 1
P2Y_2_R: P2Y2 receptor
MMP-9: Matrix metallopeptidase-9
GSK3β: Glycogen synthase kinase 3β
CCDN1: Cyclin D1
SNAIL1: Snail family transcriptional repressor 1
AP-1: Activator protein-1
SCLC: Small cell lung cancer
EMT: Epithelial–mesenchymal transition
AKR1C1: Aldo–keto reductase 1C1
CCL5: C-C motif chemokine ligand 5
CXCL9: C-X-C motif chemokine ligand 9
PD-L1: Programmed cell death-ligand 1
MHC-I: Major histocompatibility complex class I
Tregs: Regulatory T cells
JAK: Janus kinase
CAR: Chimeric antigen receptor
PKR: Protein kinase R
PD-1: Programmed cell death protein-1
HSP70: Heat shock protein 70
Wnt5a: Wnt family member 5A
CXCL5: C-X-C motif chemokine ligand 5
CXCR2: C-X-C motif chemokine receptor 2
P2X7: P2 purinergic receptors
5-FU: Fluorouracil
BRAF: B-Raf proto-oncogene
PTEN: Phosphatase and tensin homolog
MAPK: Mitogen-activated kinase-like protein
CXCR2: C-X-C motif chemokine receptor 2
cGAS: Cyclic GMP–AMP synthase
SPARC: Secreted protein acidic and rich in cysteine
Siglec-1: Sialic acid binding Ig-like lectin 1, sialoadhesin
MNS: 3,4-Methylenedioxy-β-nitrostyrene
BTK: Bruton tyrosine kinase
GSTO1: Glutathione *S*-transferase omega 1
